# Simultaneous Discovery of Positive and Negative Interactions Among Rhizosphere Bacteria Using Microwell Recovery Arrays

**DOI:** 10.3389/fmicb.2020.601788

**Published:** 2021-01-05

**Authors:** Niloy Barua, Ashlee M. Herken, Kyle R. Stern, Sean Reese, Roger L. Powers, Jennifer L. Morrell-Falvey, Thomas G. Platt, Ryan R. Hansen

**Affiliations:** ^1^Tim Taylor Department of Chemical Engineering, Kansas State University, Manhattan, KS, United States; ^2^Division of Biology, Kansas State University, Manhattan, KS, United States; ^3^Powers and Zahr, Augusta, KS, United States; ^4^Biosciences Division, Oak Ridge National Laboratory, Oak Ridge, TN, United States

**Keywords:** microdevice, microbiome, microbial communities, microbial interactions, consortia, plant growth promoting rhizobacteria, high throughput screening, microwell

## Abstract

Understanding microbe-microbe interactions is critical to predict microbiome function and to construct communities for desired outcomes. Investigation of these interactions poses a significant challenge due to the lack of suitable experimental tools available. Here we present the microwell recovery array (MRA), a new technology platform that screens interactions across a microbiome to uncover higher-order strain combinations that inhibit or promote the function of a focal species. One experimental trial generates 10^4^ microbial communities that contain the focal species and a distinct random sample of uncharacterized cells from plant rhizosphere. Cells are sequentially recovered from individual wells that display highest or lowest levels of focal species growth using a high-resolution photopolymer extraction system. Interacting species are then identified and putative interactions are validated. Using this approach, we screen the poplar rhizosphere for strains affecting the growth of *Pantoea* sp. YR343, a plant growth promoting bacteria isolated from *Populus deltoides* rhizosphere. In one screen, we montiored 3,600 microwells within the array to uncover multiple antagonistic *Stenotrophomonas* strains and a set of *Enterobacter* strains that promoted YR343 growth. The later demonstrates the unique ability of the platform to discover multi-membered consortia that generate emergent outcomes, thereby expanding the range of phenotypes that can be characterized from microbiomes. This knowledge will aid in the development of consortia for *Populus* production, while the platform offers a new approach for screening and discovery of microbial interactions, applicable to any microbiome.

## Introduction

Microbial communities are often highly diverse and have widespread impacts on human health (Clemente et al., 2012; Pflughoeft and Versalovic, 2012), agricultural productivity (Singh and Trivedi, 2017; Yadav et al., 2017), energy production (Koch et al., 2014; Doty, 2017), and water quality (Ling et al., 2018; Xia et al., 2018). Interactions among the species and strains that co-occur within microbiomes often influence their function and the establishment and success of functionally important taxa (Coyte et al., 2015). While genomic and metagenomic approaches have transformed our ability to determine community composition and species co-occurrence patterns (Barberán et al., 2012; Malla et al., 2019), understanding how interactions among strains impact community structure and function remains difficult (Foster et al., 2017; Levine et al., 2017; Ratzke et al., 2018). Despite this knowledge gap, there is a considerable need for understanding how natural community structure influences function, how communities respond to environmental pressures, and how communities can be constructed for engineered outcomes (Dodds et al., 2020). Engineered communities have provided ground-breaking approaches in a few applications, such as soil clean-up (Ojuederie and Babalola, 2017) and digestion of municipal solids (Bolzonella et al., 2005); however, the limited understanding of microbial interactions has impeded the use of synthetic communities in the majority of applications. For example, commercial development of plant growth promoting bacteria (PGPB) formulations for plant production has been limited by the fact that many useful bacterial species are incompatible with each other (Tabassum et al., 2017). These limitations require the development of new experimental tools to holistically study and understand microbe-microbe interactions (Kou et al., 2016; McCarty and Ledesma-Amaro, 2019).

The high species diversity of many microbiomes necessitates new screening tools that are designed to explore the vast number of potentially important microbe-microbe interactions. These tools must connect an observed cellular or community phenotype with genetic information from the interacting species as well as information on the interaction itself. Classical microbiological techniques for probing interactions rely on manually pairing isolates together (Goers et al., 2014), inherently low-throughput approaches that in practice are often based on qualitative observations of bulk populations. Microscale and nanoscale devices offer vast improvements by providing high-throughput measurement, observation of single cell behavior, and precise design and manipulation of the microenvironment. These approaches have advanced our understanding of microbial mutualism (Uehling et al., 2019), metabolite exchange (Burmeister et al., 2019), community adaptation to environmental pressures (Austin et al., 2010; White et al., 2019), and the role of spatial structure in driving community phenotypes (Zhang et al., 2011; Nagy et al., 2018), among other findings. Recently, Kehe et al. (2019) introduced the k-Chip, an innovative microscale platform designed to screen multi-membered communities consisting of various combinations of known isolates for emergent phenotypes. While these tools are expected to provide important advancements in our understanding of microbiomes, they are widely limited to on-chip measurements. Consequently, cells must be identified and manipulated during or prior to the screening observations, which greatly constrains both the number of strains that can be considered and undermines the ability to discover interactions involving unknown strains present in a microbiome.

**Figure 1:**
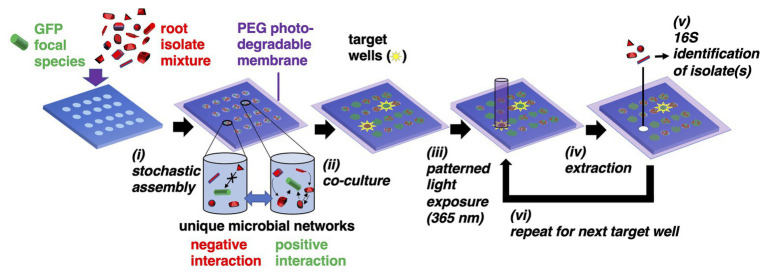
Microwell recovery arrays (MRAs) for screening microbe-microbe interactions. **(i)** Green fluorescent protein (GFP)-expressing focal species are combined with a random combination of bacteria cells from an environmental microbiome in a stochastic seeding process. Different shapes represent unique microorganisms. **(ii)** Cells are trapped within their wells using a photodegradable polyethylene glycol (PEG) hydrogel membrane and monitored in parallel during co-culture using time lapse fluorescent microscopy (TLFM). **(iii)** The membrane is ablated over a target well showing highest or lowest levels of focal species growth using patterned light exposure, then **(iv)** isolates are extracted and recovered from an opened well. **(v)** Isolates are identified using 16S amplicon sequencing. **(vi)** Steps **(iii–v)** are repeated in iterative fashion to remove each community of interest.

Here, we present the microwell recovery array (MRA), a discovery-driven, lab-on-a-chip device designed to first screen interactions within mixtures of unknown environmental isolates taken from plant root microbiomes, then uncover pair-wise or multi-species communities that best antagonize or promote the function (e.g., growth) of a non-model focal species (Figure 1). The strategy uses microwells to randomly combine the focal species – typically one with a known beneficial function (e.g., plant growth promotion) or deleterious function (e.g., pathogenesis) – with a unique sample of cells from a microbiome into an array of microwells. Our previous studies demonstrated that seeding bacteria into small (2–10 μm diameter) wells enables only small numbers of cells to be seeded into wells, where the number of seeded cells shows high dispersity across the array and follows a randomized, Poisson distribution – a process we refer to as stochastic seeding. Thus, even when a small number of unique species are present in the seeding solution, thousands of distinct, separated combinations of cells can be generated across the array for parallel observation (Hansen et al., 2016).

Here, a 10 μm well diameter was chosen to confine a small number of interacting cells together at length scales similar to those found in multi-species biofilms (Tolker-Nielsen and Molin, 2000), confinement at these length scales often facilitates inter-cellular interactions (Westhoff et al., 2020). Cells are then trapped within the wells using a previously developed photodegradable polyethylene glycol (PEG)-based membrane (van der Vlies et al., 2019), co-cultured, and then focal strain growth in each well is tracked with time lapse fluorescent microscopy (TLFM). Cellular communities showing a desired phenotype (e.g., highly enhanced or diminished focal species growth) can be extracted from any individual well using a patterned light source to spatially ablate the membrane, releasing cells into solution for recovery. The extraction and recovery capabilities are the key enabling features of the platform, allowing for sampling of a microbial community from any number of individual microwells that indicate a desired outcome, in a sequential fashion. Ultimately, this allows one to identify the interacting strains after the screening step. Relying on the stochastic seeding to generate randomized combinations between multiple species, thousands of distinct combinations of cells can be observed in a single screen. Extraction also enables follow-up phenotypic characterization with standardized assays to confirm the interaction.

To develop the approach, we first investigate co-culture in the MRA format using a well-characterized interaction between *Pseudomonas aeruginosa* and *Agrobacterium tumefaciens* (An et al., 2006; Ma et al., 2014), followed by a microbiome screen using *Pantoea* sp. YR343 as the non-model focal species. Strain YR343 is a Gram-negative, plant growth promoting bacterium (PGPB) isolated from the rhizosphere of an eastern cottonwood *Populus deltoides* tree (Bible et al., 2016; Estenson et al., 2018). As *Populus* trees are promising biofuel feedstocks (Polle et al., 2013), uncovering interactions that influence the function of beneficial organisms in its rhizosphere has received intensive interest in recent years (Hacquard and Schadt, 2015; Utturkar et al., 2016; Blair et al., 2018; Veach et al., 2019). However, due to the fact that the *Populus* root microbiome is highly complex and diverse, many microbe-microbe interactions are unknown (Beckers et al., 2017). *Pantoea* sp. YR343 can also colonize *Triticum aestivum* and stimulate lateral root formation (Bible et al., 2016; Estenson et al., 2018). Likewise, related *Pantoea* strains have garnered interest for antibiotic production (Sammer et al., 2012), bioremediation and waste recycling (Zhu et al., 2008), and cancer treatment (Dutkiewicz et al., 2016b). On the other hand, other *Pantoea* sp. can be pathogenic in plant, animal, and human systems (Dutkiewicz et al., 2016a). Thus, uncovering unique sets of organisms that both promote or inhibit *Pantoea* growth, as demonstrated here, has use in several contexts.

## Materials and Methods

### Preparation of Bacteria Strains and *Populus trichocarpa* Samples

Bacteria strains and plasmids used are listed (Supplementary Table 1). We introduced pSRKKm-sfGFP into *A. tumefaciens* C58 and pSRKKm-mcherry into *P. aeruginosa* PAO1 *via* mating with *Escherichia coli* S17-1 λpir carrying the respective plasmids using previously described methods (Morton and Fuqua, 2012). These plasmids were transformed into competent S17-1 λpir *E. coli* strains using calcium chloride heat-shock transformation. *Pantoea* sp. YR343-green fluorescent protein (GFP) constitutively expresses EGFP from a chromosomal insertion as previously described by Bible et al. (2016). All strains and isolates used were stored in 25% glycerol at −80°C. Further information on *A. tumefaciens* C58, *P. aeruginosa* PAO1, and *Pantoea* sp. YR343 culture is included in Supplementary Information. For extraction of microbes from *Poplar* root, a sample of Nisqually-1 *Populus trichocarpa* root was first obtained from the greenhouse facilities at Oak Ridge National Laboratory. Roots were removed from soil and the aerial parts of the plant were separated from the root system. Large soil aggregates were removed by manually shaking by hand. The remaining portions of the roots were removed with sterile blades. Root pieces were then washed extensively with 1.5 L of sterile ice-cold PBS-tween20 solution (7 mM Na_2_HPO_4_, 3 mM NaH_2_PO_4_, pH 7.0, and 0.05% tween20). The washed solution was filtered through 0.45 μm sterile syringe filters (Whatman) to remove larger particles in the suspension. The filtered solution was centrifuged for 15 min at 4,400 rpm to obtain the pellet containing rhizosphere-enriched isolates (de Souza et al., 2016). Glycerol stocks were prepared for *P. trichocarpa* root isolates and stored frozen at −80°C. Bacterial cells were later revived by scrapping off a small amount of frozen cells using a sterile inoculation loop and mixing in 2 ml R2A broth media (pH: 7.2 ± 0.2, Teknova) in sterile test tubes and cultured for 24 h (28°C, 215 rpm). The community composition of both the rhizosphere-enriched isolate sample and the R2A media culture used to seed the microarray were analyzed using 16S ribosomal RNA (rRNA) community analysis (Supplementary Text, Supplementary Figure 1).

### MRA Design and Fabrication

Microwell recovery arrays were designed to contain 10 μm diameter microwells etched to 20 μm well depths, spaced at a 30 μm pitch. The array consisted of a 7 × 7 grid of sub-arrays, each sub-array contained a 15 × 15 array of microwells, totalling 11,025 microwells available for analysis. Each well in the 15 × 15 sub-array was assigned with its own unique on-chip address for identification using brightfield microscopy (Supplementary Figure 2). Microwell arrays were fabricated on 3-inch diameter N-type silicon wafers (University Wafers) after coating with a 1 μm thick layer of Parylene N (PDS 2010 Labcoater, Specialty Coating Systems Masigol et al., 2018). Arrays were then fabricated in a cleanroom environment using photolithography (Supplementary Figure 3) following previous protocols (Hansen et al., 2016; Masigol et al., 2017).

### Bacteria Seeding and Trapping on Microwell Arrays

C58-GFP and PAO1-mCherry were grown in LB, and YR343-GFP was grown in R2A media to mid-log phase and then resuspended in their respective growth media to an OD_600_ of 0.2. To inoculate microwell substrates, 700 μl of this cell suspension was then incubated over an individual MRA substrate (Supplementary Figure 2) at room temperature for 1 h. The substrates were dried and the parylene was peeled off of the microwell surface along with the cells attached to the background regions of the array by applying Scotch tape and forceps (Hansen et al., 2016). For studies involving C58-GFP and PAO1-mCherry co-culture, the seeding solution contained C58-GFP and PAO1-mCherry cells in a 1:1 or 1:100 ratio at a total OD_600_ of 0.1. For studies involving YR343-GFP and *P. trichocarpa* rhizobiome co-culture, washed YR343-GFP cells and *P. trichocarpa* rhizobiome cells were mixed to achieve a YR343-GFP:isolate ratio of approximately 1:100 in the seeding solution at an OD_600_ of 0.2. To keep the cell concentrations of C58-GFP in co-culture experiments constant, PAO1-mcherry at OD_600_ = 10 was added to C58-GFP at OD_600_ = 0.1 to reach a C58-PAO1 ratio of 1:100. The inoculum was then diluted to OD_600_ = 0.1 and 700 μl of this inoculum was then seeded into microwell array substrates as described above. Similarly, OD_600_ = 0.2 cultures of *P. trichocarpa* rhizobiome was mixed with OD_600_ = 20 of YR343-GFP to reach a YR343-*P. trichocarpa* ratio of 1:100. This seeding suspension was diluted to OD_600_ = 0.2 and seeded on top of microwell arrays for co-culture studies. For YR343-*P. trichocarpa* studies, the photodegradable membrane was then attached to the seeded array (van der Vlies et al., 2019). A schematic describing the seeding and trapping steps is provided (Supplementary Figure 4).

### Time Lapse Fluorescence Microscopy

A Nikon Eclipse Ti-E inverted microscope with NIS Elements software, a motorized XYZ stage, a humidified live-cell incubation chamber (Tokai Hit), and a DS-QiMc monochromatic digital camera was used for TLFM measurements. Seeded microwell arrays (with or without the photodegradable membrane) were attached to an LB-agar PDMS coverslip (Supplementary Figure 4). PDMS was required to enable sufficient oxygen diffusion into the wells during, prior experiments using glass coverslips resulted in poor culture for aerobic bacteria due to limited oxygen diffusion (Hansen et al., 2021). The substrate was then placed in a custom 3D printed scaffold designed to accommodate the microwell array while submerged under liquid media. The scaffold aided in image acquisition by maintaining a constant distance (100 μm) between the array and the glass slide, enabling the microwell substrate to stay within the focal plane during the culture period (Supplementary Figure 5). More information on microwell attachment to these materials and on the design of the scaffold can be found in the Supplementary information. The scaffold along with the inverted microwell substrate were then placed inside a humidified live-cell incubation chamber at 28°C for imaging. A FITC filter was used to image C58-GFP strains (20×, 200 ms, and 17.1 × gain) and a TRITC filter was used to image PAO1-mCherry strains (20×, 300 ms, and 17.1 × gain). For YR343-GFP, images were taken with a FITC filter (20×, 300 ms, and 36× gain) with a neutral density filter with 25% standard light intensity to minimize photobleaching. With these imaging conditions, individual cells within the wells could be resolved. Brightfield images were also taken at each section of the array after fluorescent imaging. Images of the microwell arrays were taken every 60 min during culture. Green and red fluorescent images from the C58-GFP and PAO1-mCherry co-culture system were analyzed using Protein Array Analyzer tool in ImageJ to generate growth profiles for each organism. YR343-GFP in monoculture or mixed culture was evaluated using an image analysis routine in MATLAB to identify wells with highest and lowest growth levels for extraction.

### Image Analysis

Time-lapse fluorescent microscopy and fluorescence-based image analysis can be routinely used to generate and access bacteria growth trajectories in this microwell format, as recently described by Timm et al. (2017) ImageJ was used to quantify growth trends of the C58-GFP and PAO1-mCherry. MATLAB was used to identify wells with highest and lowest levels of growth for YR343-GFP monoculture and co-culture studies. Here, simultaneous brightfield and fluorescence images of each array subsection consisting of 15 × 15 microwells were taken every hour for a 15 h culture period. Brightfield and fluorescence images were imported and sorted based on subarray location, then the location of the wells was recorded and fluorescence intensities were averaged across each individual well and subtracted from background levels for each time point. Average growth rates and end point well intensities were then quantified across the entire microwell population. Outlier wells with highest levels of deviation in end-point fluorescence (*t* = 12 h) were identified as target wells using the Grubb’s outlier test (Grubbs, 1950) and their addresses were recorded. From these outlier wells, the top 5 growth promoting wells with highest average growth rates and top 4 antagonist wells with the lowest average growth rates were picked for extraction. In addition, four wells with nominal average growth rates were picked for extraction.

### Recovery of Isolates From Wells and Isolate Naming Convention

The extraction procedure was slightly modified from van der Vlies et al. (2019), which was previously developed for highly efficient removal of cells from individual wells with minimal cross-talk, thus offering well-specific extraction and is described in Supplementary Information. Extraction occurred from the microwell array in a sequential fashion, first from five different target wells in which YR343-GFP exhibited promoted growth (P1–P5), then four different target wells in which YR343-GFP exhibited antagonized growth (A1–A4), and finally four different target wells in which YR343-GFP exhibited intermediate growth (N1–N4). Extracts from each well were plated onto solid R2A media (28°C, overnight) for recovery. After culture, five distinct isolates (A–E) were picked based on unique colony morphology and streak purified. Isolates in Supplementary Figure 6 are thus labeled according to the microwell they were isolated from, then the order at which it was extracted from the array, then the order at which it was picked from the plate after recovery. For example, isolate A4A, the isolate that most strongly antagonizes YR343 growth, was extracted from the fourth antagonistic well and was the first colony picked from the R2A plate. Following extraction, extract containing the suspension of cells from an individual microwell was plated onto R2A media. Colonies were again cultured in liquid media overnight (28°C, 215 rpm) and stored in glycerol stocks at −80°C.

### Identification With 16S rRNA Sequencing

Individual colonies were cultured in R2A media and genomic DNA of each isolate was extracted using the Promega (Madison, WI) Wizard® DNA Purification kit, diluted to 20 ng/μl in 20 μl aliquots and sent to Genewiz (South Plainfield, NJ, United States) for 16S rRNA Sanger sequencing of the V1–V9 regions, enabling identification with approximately genus-level specificity. The sequences were aligned using MUSCLE (Edgar, 2004) and generated a maximum likelihood phylogenetic tree based on partial 16S rRNA sequences (1,007 bp) using PhyML 3.3.20190909 (Guindon et al., 2010) with 1,000 bootstrap replicates and using the Smart Model Selection (Lefort et al., 2017) tool based on Akaike Information Criterion, a starting tree estimated using BIONJ, and the NNI method for tree topology improvement.

### Validation Using 96-Well Plate Cultures

To obtain cell free culture fluid (CFCF) from individual isolates, each isolate was cultured (28°C, 3,000 rpm) in 2 ml of R2A broth media overnight, and then cells were removed from the media by centrifugation (2,000 g, 10 min). To obtain CFCF from combinatorial mixtures, isolate panels were inoculated individually in R2A media and cultured overnight, followed by cell removal by centrifugation. CFCF from each isolate was then mixed together at equal volumes to obtain combinatorial CFCF. To obtain conditioned media, isolate or combinatorial CFCF was mixed with YR343-GFP in fresh R2A media at a 1:1 volumetric ratio to reach an initial OD_600_ value of 0.1 (final volume = 100 μl), at which point growth was quantified with a Biotek Epoch 2 Multi-Mode Microplate Reader (28°C, 300 rpm). Unconditioned media (UCM) were obtained following the same procedure except 1X PBS was added to fresh R2A media instead of isolate CFCF. To verify the OD_600_ measurement was due to YR343-GFP growth, CFCF from selected isolates without inoculation of YR343 was also measured. A total of *n* = 6 independent replicates were measured for each culture condition. Growth rates and carrying capacities of each condition were quantified using Growthcurver (Sprouffske and Wagner, 2016) and compared using the Wilcoxon two-sample test.

## Results and Discussion

### Microwell Recovery Arrays Enable Parallel Monitoring of Microscale Co-culture Sites and Generation of Outlier Wells With Unique Growth Phenotypes

Our prior results demonstrated that microwell arrays could be used for parallel tracking of the growth of *P. aeruginosa* PAO1 communities during mono-culture in microwells, where small (5–10 μm diameter) wells were used to generate high variations in inoculum densities across the array during the seeding step, and growth outcomes were dependent on inoculum density and the level of spatial confinement present (Hansen et al., 2016). To develop the platform for multi-species co-culture, here, we added *A. tumefaciens* C58 to this system. Strains PAO1 and C58 have a well-characterized, competitive interaction *in vitro*, where PAO1 tends to outcompete C58 due to quorum sensing-regulated growth rate and motility advantages (An et al., 2006; Ma et al., 2014). A mixture of C58 cells expressing GFP (hereafter C58-GFP) to PAO1 cells expressing mCherry protein (hereafter PAO1-mCherry) was inoculated into 10 μm diameter wells at a seeding concentration of OD_600_ = 0.1. Based on our previous characterizations (Hansen et al., 2016), we estimate that this results in ~20 cells per well. Under these conditions, PAO1-mCherry and C58-GFP cells are paired together at a high-dispersity due to the stochastic, Poisson seeding process (Hansen et al., 2016). C58-GFP:PAO1-mCherry seeding ratios of 1:1 and 1:100 were both investigated.

**Figure 2:**
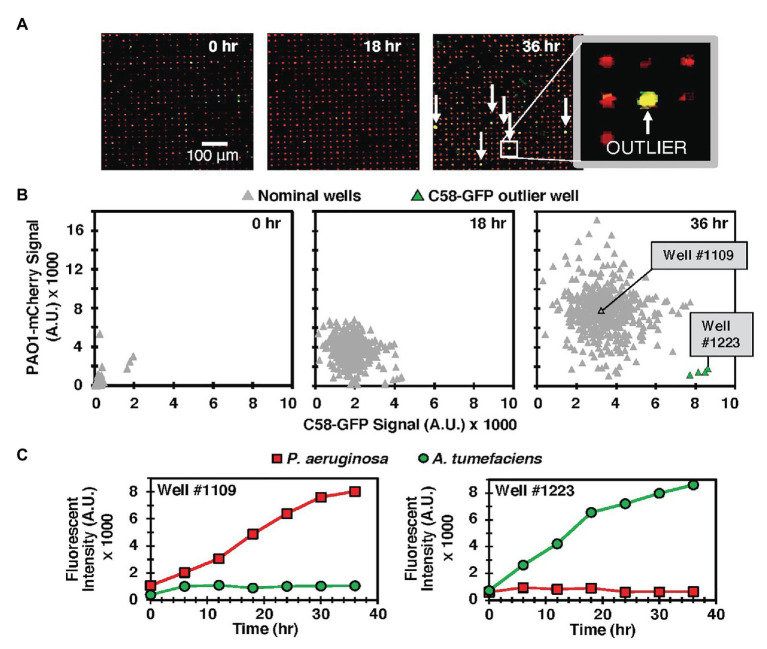
**(A)** Model C58-GFP (green) – PAO1-mCherry (red) co-culture in the MRA. Arrows indicate rare outlier wells where C58-GFP outgrew PAO1-mCherry. **(B)** Scatter plot of green (C58-GFP) vs. red (PAO1-mCherry) well signals from a sample 549 well array at various time points. Outlier wells where C58 outgrew PAO1 are identified after the culture period (green). **(C)** Individual growth trajectories from a sample nominal well (well #1109), where PAO1 growth rate was significantly higher than that of C58 and an outlier well (Well #1223), where C58 outgrew PAO1.

We observed similar qualitative outcomes at both seeding ratios. In each case, the MRA platform enabled parallel tracking of species growth according to the respective fluorescence emission signals from each addressable well during co-culture and end-point growth levels as well as signature growth profiles could be attained from each well with image analysis (Figure 2, Supplementary Figure 7). For the 1:100 seeding ratio, which was the seeding ratio used in the following studies, a comparison of end-point fluorescence signals after a 36 h co-culture period identified that the majority of the wells (96%) generated outcomes where PAO1-mCherry outgrew C58-GFP (Figures 2B,C). This was likely because of a favorable PAO1 seeding ratio, PAO1 growth advantages, or a combination of both factors. However, in the MRA format, outlier testing identified a minority (4%) of wells with communities dominated instead by C58-GFP cells after co-culture (Figures 2B,C). This finding reveals that high-dispersity microbe pairing between competing species produces wells with rare growth outcomes after co-culture. Here, despite C58 cells being present at lower concentrations in the seeding solution, the stochastic seeding process generated a minority of wells with conditions allowing C58-GFP to grow well. This finding was leveraged toward more complex co-culture systems, where the stochastic seeding and parallel growth tracking features of the MRA are applied to screening interactions in environmental microbiomes.

### Co-culture of *Pantoea* sp. YR343 With Stochastically Assembled Communities From the *P. trichocarpa* Rhizosphere Simultaneously Generates Positive and Negative YR343 Growth Outcomes

To extend the microwell platform capabilities to screening non-model test species against unknown isolates, we screened rhizobiome samples from the *P. trichocarpa* root microbiome for effects on the growth of focal species *Pantoea* sp. YR343 expressing GFP (hereafter, denoted YR343-GFP). Here, we used stochastic seeding, attachment of the photodegradable PEG membrane (van der Vlies et al., 2019), and focal species growth monitoring to identify rare combinations of cells generating unique YR343-GFP growth profiles (Figure 1, Steps i and ii). To characterize the composition of the seeding solution, 16S community analysis was used (Supplementary Figure 1) and we observed 120 OTUs from the root washing and 85 OTUs after culturing the root washing in R2A media to prepare the isolate mixture used to seed the wells. Thus, it was expected that the YR-343 focal species is combined with random samplings of cells belonging to 85 different OTUs in wells throughout the array.

**Figure 3:**
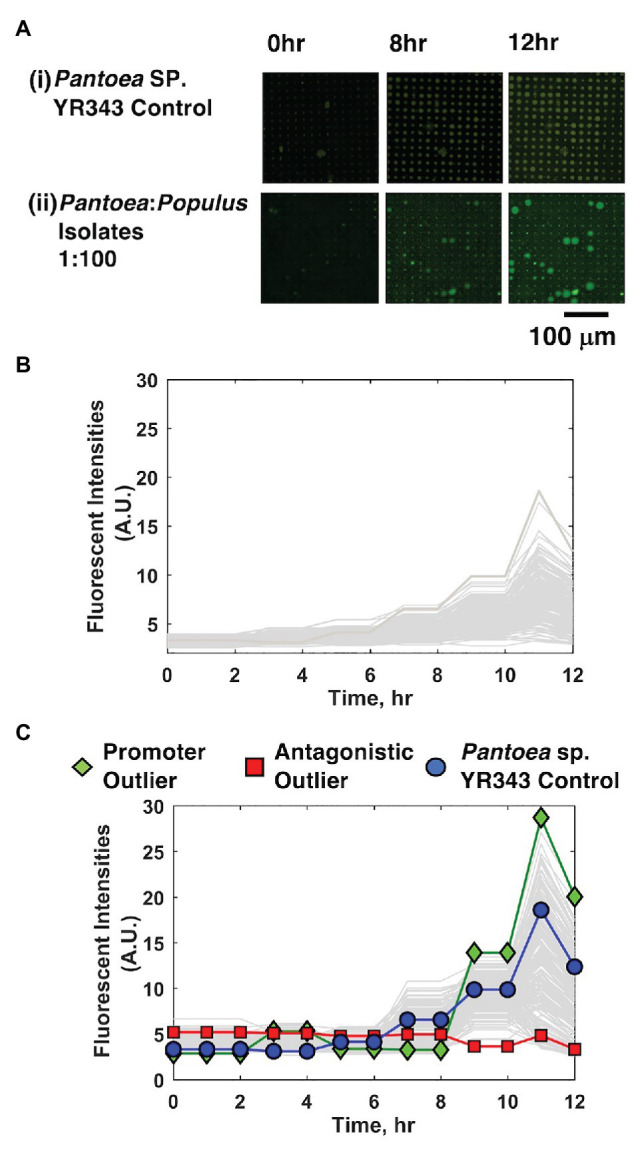
YR343-GFP growth in mono-culture and co-culture within 10 μm microwells. **(A)** TLFM images of a sample 15 × 15 array of microwells after **(i)** seeding only YR343-GFP or **(ii)** seeding YR343-GFP with isolates from a *Populus trichocarpa* rhizobiome. **(B)** Growth curves generated from a sample 900 microwell array during YR343-GFP mono-culture, or **(C)** YR343-GFP co-culture with rhizosphere isolates. Outlier wells representing growth promoting and antagonistic communities, respectively were identified from the growth curves.

Cell mixtures were seeded into 10 μm diameter microwells at high density (OD_600_ = 0.2) and at a 1:100 YR343:isolate ratio, cultured, and growth kinetics in each well were tracked over the course of 12 h using TLFM. Based on prior results (Hansen et al., 2016), we estimate this seeding condition generates ~40 cells/well. The 1:100 seeding ratio follows from the previous system and ensures that the focal species will be combined with several unknown isolates in each well during the co-culture. For comparison, monoculture arrays consisting of only YR343-GFP focal species were used as a control. In each case, YR343-GFP growth was evaluated in 225 co-culture microwells from a 15 × 15 well grid (Supplementary Figure 2) across 16 selected arrays on a single substrate (*n* = 3,600 microwells total). Here, R2A media were chosen as a generalist culture media. This media have been used to recover more than 300 phylogenetically diverse isolates from *P. trichocarpa* rhizosphere and endosphere samples, and so should permit co-culture of a large number of combinatorial strain mixtures within the microwell environment (Blair et al., 2018). While the YR343-GFP monoculture generated growth profiles across the array with relatively low variance (*σ*^2^ = 3.55) according to final end-point fluorescence levels, mixed cultures generated a wider range of growth profiles, with final growth levels of higher variance (*σ*^2^ = 17.55), indicating an impact due to the addition of the environmental isolates (Figures 3A–C).

In co-culture, 14% of the wells contained microcolonies that appeared to grow out of the wells and into the membrane space, causing the microcolony diameter to expand beyond the well diameter (>10 μm; Figure 3Aii), indicating a positive interaction. While the locations on the array where this effect occurred appeared random, we checked for the possibility of crosstalk between neighboring wells, where a developing microcolony may influence growth in another well due to diffusion of metabolites or other biomolecular products. Of wells with this enhanced growth phenotype, 2% of neighboring wells also showed this phenotype, suggesting that well-to-well crosstalk can occur. The possiblity of falsely identifying an interaction due to well-to-well crosstalk is accounted for with follow-up, off-chip validation experiments that verify the interaction after it is found in the inital screen (described in Section “Interactions Can Be Recapitulated in 96-Well Plate Format for Validation”). On the other end, wells showing decreases in well fluorescence signal were also identified, these decreases were caused by lysis of the focal species and GFP diffusion from the wells, as previously observed when using PAO1 as the focal species (Hansen et al., 2016). This effect was noted in 34% of co-culture microwells. Wells that initially contained a fluorescent signal above background levels, followed by highest decreases in fluorescence signal were identified as containing candidate cells antagonistic to YR343-GFP. This ensured that these wells initially contained the focal species, and that its growth was inhibited during co-culture. The rest of the wells did not show evident increases or decreases in YR343-GFP growth.

### Sequential Extraction, Recovery, and Identification of Isolates From Microwell Communities

**Figure 4:**
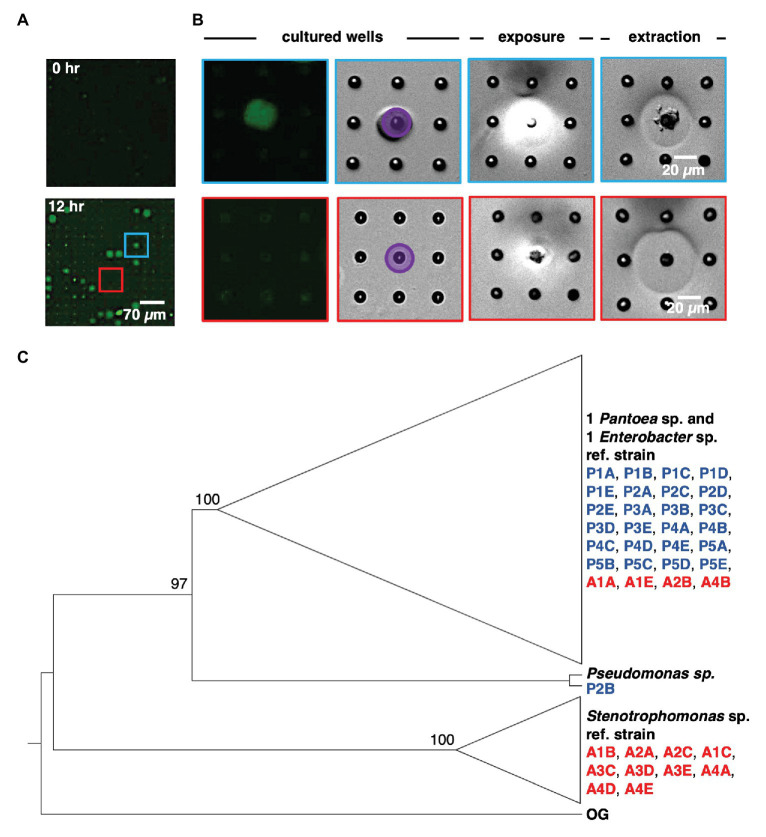
Sequential removal of growth-promoting and antagonistic communities from an array sub-section after co-culture. **(A)** Microwell array before and after co-culture. This 15 × 15 microwell array contained both a YR343 growth promoting community (blue) and YR343 antagonistic (red) community that were targeted for extraction. **(B)** Targeted removal of the microwell community in which YR343 grows to its highest observed end-point fluorescence (top row and blue outline), followed by targeted removal of a microwell community in which YR343-GFP grew poorly (bottom row and red outline). Purple area denotes UV exposure area used for membrane degradation. **(C)** Maximum likelihood phylogenetic tree based on partial 16S ribosomal RNA (rRNA) sequences (1,007 sites) of select reference strains and isolates extracted from promoted (P) and antagonized (A) wells. We collapsed the branches of the monophyletic group composed of *Enterobacter* sp. and *Pantoea* sp. strains and the clade of *Stenotrophomonas* sp. strains. *Agrobacterium tumefaciens* C58 was used as the outgroup (OG) organism and the following reference strains were included: *Pantoea* sp. YR343, *Enterobacter cloacae* E3442, *Pseudomonas putida* S13.1.2, and *Stenotrophomonas maltophilia* NCTC10259. We labeled nodes with corresponding bootstrap percentages.

Following on-chip analysis in mixed culture arrays, the patterned illumination tool was used to extract communities from the five wells with highest fluorescence signal after 12 h of culture (Figures 4A,B). This was followed by extraction of communities from four wells with the lowest levels of YR343-GFP growth, and four wells where YR343-GFP grew to intermediate levels. Extraction required exposure of a patterned 365 nm light (20 mW/cm^2^, 10 min) to remove the membrane over the well. While 365 nm light has the potential to damage bacteria, these exposure conditions were previously found suitable for retrieving viable bacteria from wells (van der Vlies et al., 2019). Membrane removal was confirmed by brightfield microscopy, at which point cellular material was observed moving out of the wells and into solution (Figure 4B). After exposure, arrays were washed with extraction buffer (R2A media + 0.05% Tween20 solution) to retrieve cells from an opened well. Extraction buffer was then plated onto R2A-agar for growth and recovery of individual colonies. During our previous characterizations of this procedure, we noted that >99.9% of bacteria originated from opened wells as opposed to outside contamination (van der Vlies et al., 2019), which provided high confidence that the recovered product here originated from the target well. We also previously observed that bacteria could be completely removed from wells after washing (van der Vlies et al., 2019), thus we expected minimal cross-contamination when opening additional wells for further sampling. After recovery, phylogenetic analysis based on 16S rRNA sequences of all strains isolated from each targeted microwell was performed (Supplementary Figure 6). The analysis revealed that all extracted microwells identified as growth promoting for YR343-GFP (five of five) harbored *Enterobacter* sp./*Pantoea* sp. strains, and one of these wells contained at least one *Pseudomonas* sp. strain. In stark contrast, all wells identified as antagonistic to YR343-GFP contained at least one *Stenotrophomonas* sp. strain. Several of these wells (three of four) also contained at least one *Enterobacter* sp. or *Pantoea* sp. strain (Figure 4C). All isolates obtained from the wells with nominal effects on YR343-GFP are phylogenetically related to *Enterobacter* sp. and *Pantoea* sp. strains. Given that the extraction method was efficient and specific for recovering cells from the targeted wells (van der Vlies et al., 2019), the recovered isolates were expected to be responsible for the promoting or antagonistic effects on YR343, but required validation with an independent, off-chip test.

### Interactions Can Be Recapitulated in 96-Well Plate Format for Validation

The extraction of cellular communities from the MRA allows for off-chip validation and characterization of the microbial interactions observed during the screen. This capability is critical for validation, as the high density of microwells (625 wells/mm^2^) has potential to cause false positives, perhaps due well-to-well cross-talk due to diffusion of molecules. This necessitates that the interactions observed in the screen are also observed in an independent validation assay. To address this, we used a 96-well plate format to measure how strains isolated from MRA influenced the growth of YR343-GFP. This represented a scaled-up environment (from 1.6 pL microwell volumes to 100 μl solution volumes) that precludes diffusive crosstalk from neighboring wells.

**Figure 5:**
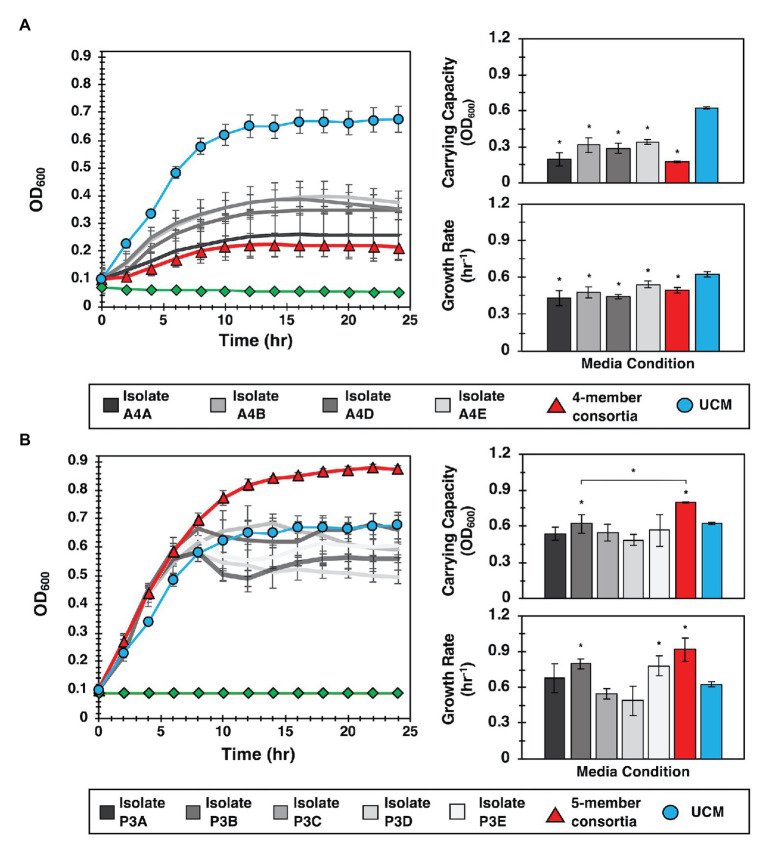
Interactions identified in the MRA can be validated in 96-well plate format. **(A)** Left: YR343 growth curves after inoculation into conditioned media from the antagonistic isolate, the isolate consortia, or unconditioned media (UCM). The control (green line) is conditioned media that was not inoculated with YR343 to verify that there was no growth carry over or contaminating microbes present. Right: Corresponding carrying capacity and growth rates for each growth curve. **(B)** Left: Analogous YR343 growth curves after inoculation into conditioned media from a promoter isolate or the promoter isolate combination. Right: Corresponding carrying capacity and growth rates. All growth experiments occurred at 28°C, 215 rpm. Statistical differences were identified by comparison of growth metrics between YR343 culture in conditioned media from each isolate or isolate mixture and YR343 growth in UCM (Wilcoxon two-sample test, ^*^p < 0.01, *n* = 6 independent experiments).

For these evaluations, we hypothesized that both growth promotion and inhibition measured in MRA format resulted from diffusive interactions between the focal species and the collection of isolates present within a well. To test this hypothesis, YR343-GFP was cultured in 96-well plate format in media conditioned by four selected isolates recovered from a selected antagonist well (Well A4, Supplementary Figure 6). Conditioned media were obtained by first culturing isolates in R2A media to stationary phase, then removing the cells to obtain CFCF. Fresh R2A media was then added to the CFCF in a 1:1 volumetric ratio to supply growth nutrients, and YR343-GFP was inoculated for growth monitoring. Conditioned media obtained using CFCF from a combined co-culture of all 4 antagonistic strains was also evaluated. These growth curves were compared to a control curve with YR343-GFP growth in UCM, which consisted of R2A media instead supplemented with blank 1X PBS buffer at the same volumetric ratio. A second control curve consisting of conditioned media without YR343 inoculum was also included to verify that measured growth was not due to contaminating microbes. Growthcurver R was then used to estimate bacterial carrying capacity and growth rate (Sprouffske and Wagner, 2016) in each experiment (Supplementary Figure 8). Congruent with microwell observations, we observed that conditioned media from 4 isolates significantly reduced the carrying capacity and growth rate of YR343-GFP compared to its culture in UCM (Figure 5A). Conditioned media from the combined 4-member antagonist combination also showed significantly lower carrying capacity and growth rate compared to the unconditioned control media (Supplementary Tables 2, 3). CFCF from *Stenotrophomonas* isolate A4A had statistically equivalent growth metrics as that from the CFCF consortia, suggesting that this strain is the most potent inhibitor of YR343.

To investigate the effect of the strains identified as YR343 growth promoters, YR343-GFP growth was again monitored in media conditioned with CFCF from clonal cultures, here using five isolates selected from a selected promoter well (Well P3, Supplementary Figure 6). To test for an emergent effect, additional control curves from conditioned media containing CFCF produced from a co-culture of the combined five isolates was also evaluated. When YR343 growth in conditioned media from the CFCF of individual isolates was measured, only two were able to increase growth rate and one was able to increase carrying capacity (Figure 5B; Supplementary Figure 9). Strikingly however, the CFCF from the five-member consortia was able to provide highest increases in both YR343 growth rate and carrying capacity. The five-member consortia also provided a statistically significant increase in carrying capacity compared to isolate P3B, the individual isolate that generated the highest increase in YR343 carrying capacity after conditioning media on its own (Supplementary Tables 4, 5). To further verify that antagonistic or promoting behavior was unique to the strains isolated from the promoting and inhibitory microwells, the same 96-well plate analysis was performed using nominal isolates taken from a microwell that showed intermediate growth of YR343-GFP during on-chip co-culture. This served as a final control to verify the 96-well plate assay accurately recapitulates growth behavior observed on the MRA. Here, a well with final endpoint growth level comparable to YR343 monoculture was identified, and four isolates were extracted from the well. YR343-GFP growth was then monitored in media conditioned with CFCF from clonal cultures and the combined four-member consortia cultures. YR343 growth in conditioned media from the CFCF of individual isolates and the CFCF from the four-member consortia did not provide significant increases or decreases in YR343 growth rate or carrying capacity (Supplementary Figures 10, 11; Supplementary Tables 6, 7).

Taken together, these findings indicate that the observed enhancement of YR343’s population growth corresponds with the behavior observed in the microwell environment, and that in some cases, it can depend on the presence of multiple strains, not simply the consequence of a pairwise interaction. As such, the enhanced YR343 growth is an emergent property of the community of species recovered using the MRA, demonstrating the unique power of this approach to identify functions dependent on higher-order interactions among bacterial species.

## Conclusion

The MRA examines thousands of combinatorial unique, multi-species communities to discover both antagonistic and growth promoting interactions on a focal species. Using this new approach, we simultaneously identified individual strains that antagonize focal species growth, as well as multi-strain consortia that uniquely promotes focal species growth only when co-cultured in combination. The platform is the first of its kind, unique because it (i) screens organisms that are unknown during the screening step, dramatically expanding the number of interactions and cellular combinations that can be accommodated, and (ii) screens in combinatorial fashion to uncover higher-order microbial networks that generate emergent phenotypes, which cannot be measured with other platforms or devices. The platform allows for the user to perform the co-culture in a defined culture medium, which must be carefully selected based on the question or goal of the screen. The key innovation underlying this capability is the ability to recover cells from specific microwells of interest, thereby allowing for subsequent off-chip genetic characterization for species identification then phenotypic characterization for validation of the interaction. This enables one to input any number of bacteria strains into the device for analysis. Extraction then enables one to streamline the screen with established techniques, such as -omic based analysis of samples and follow-up validation of the uncovered interactions using standardized methods, as demonstrated in this work. In our laboratory, MRA fabrication and materials cost ~$15 per screen, which compares favorably to other comparable techniques such as flourescence-activated cell sorting (FACS), which often has a higher associated cost ($100–200/h) and is not directly amenable to a co-culture format. The improved throughput at which different interactions can be tested also provides a significant saving in both time and effort.

For the first generation of the MRA, we have developed its use toward screening interactions that influence growth phenotypes. A drawback of the current platform is that it screens interactions based on growth in an environment that is both chemically and physically different than the rhizosphere, thus interactions that are identified in the MRA must still be evaluated in the relevant natural enviornment (e.g., *in vivo*). Also, the MRA requires that the interacting isolates are also culturable in the media added, limiting the number of interactions that can be accounted for. Finally, the user should excersize caution when extracting cells using the 365 nm light source, as this wavelength can have a bactericidal effect. If direct UV exposure is a concern, the pattern of light can be varied to expose only the edges or sides of the wells, which is also effective in releasing cells from wells while minimizing light exposure (van der Vlies et al., 2019). Despite these current limitations, the MRA approach has potential to be expanded toward screening microbiomes for organisms that have positive or negative effects on other focal species functions, provided that the function can be coupled to a fluorescence reporter (e.g., a GFP promoter-reporter). This may include microbial interactions that affect quorum sensing activation (Abisado et al., 2018), virulence factor expression (Rutherford and Bassler, 2012), and plasmid conjugation (Tecon et al., 2018), to name a few. While demonstrated here for the *P. trichocarpa* root microbiome, the platform is directly amenable to screening interactions across any microbiome where high species diversity is present, which may include the gut, soil, freshwater and marine ecosystems, and other rhizosphere environments.

## Data Availability Statement

The raw data supporting the conclusions of this article will be made available by the authors, without undue reservation, to any qualified researcher.

## Author Contributions

NB, AH, RP, TP, and RH: conceived and designed experiments. NB, AH, and KS: performed experiments. SR, RP, and JM-F: developed or contributed analysis tools/reagents/materials. NB, AH, TP, and RH: wrote the paper. All authors contributed to the article and approved the submitted version.

### Conflict of Interest

RH and TP have filed a patent application on this technology. RP and SR were employed by the company Powers & Zhar.

The remaining authors declare that the research was conducted in the absence of any commercial or financial relationships that could be construed as a potential conflict of interest.
