# Supplementary material for: Simultaneous Discovery of Positive and Negative Interactions Among Rhizosphere Bacteria Using Microwell Recovery Arrays
Source: Front Microbiol. 2021 Jan 5;11:601788. doi: 10.3389/fmicb.2020.601788 (PMC7813777; doi:10.3389/fmicb.2020.601788)
Supplement: Supplementary file 1 [file Data_Sheet_1.pdf]

Supplementary Material

Simultaneous Discovery of Positive and Negative Interactions Among Rhizosphere Bacteria Using Microwell Recovery Arrays

Author names and affiliations: Niloy Barua[1], Ashlee M. Herken[2], Kyle R. Stern[1], Sean Reese[3], Roger L. Powers[3], Jennifer L. Morrell-Falvey[4], Thomas G. Platt[2*], Ryan R. Hansen[1*]

[1]Tim Taylor Department of Chemical Engineering, Kansas State University, Manhattan, Kansas, United States.

[2]Division of Biology, Kansas State University, Manhattan, Kansas, United States.

[3]Powers and Zahr, Augusta, Kansas, United States.

[4]Biosciences Division, Oak Ridge National Laboratory, Oak Ridge, Tennessee, United States.

*** Correspondence:**
Thomas G. Platt
Email: tgplatt@ksu.edu

Ryan R. Hansen
Email: rrhansen@ksu.edu

This PDF file includes:

Supplementary text

Figures S1 to S11

Tables S1 to S7

SI Reference

1 *P. aeruginosa* and *A. tumefaciens* culture

A model system comprised of *Agrobacterium tumefaciens* C58 expressing GFP (C58-GFP) and *Pseudomonas aeruginosa* PAO1 expressing mCherry (PAO1-mCherry) was used to characterize seeding behavior and interaction in microwell recovery arrays (MRAs). Glycerol stocks were prepared for both strains and stored at -80°C. C58-GFP and PAO1-mCherry were cultured on LB-agar for 24 hrs at 28 and 37°C respectively. For liquid culture of C58-GFP and PAO1-mCherry, a single colony was picked using a sterile inoculation loop, inoculated in 2 mL LB media (10g BactoTryptone, 5g Yeast, 10g NaCl and 15g agar per 1000mL) in sterile test tubes and cultured in a shaker (28 and 37°C respectively, 215 rpm) for 24 hrs. 150 µg Kanamycin and 0.5 µg IPTG (Sigma) were added to each ml of the liquid culture for both C58-GFP and PAO1-mCherry cultures. The tubes were then centrifuged (Eppendorf Centrifuge 5702 Series Ea) and the media was changed every 24 hrs for up to 1 week to keep cells viable.

2 Growth studies of *Pantoea* sp. YR343 monoculture

For solid phase cultures, *Pantoea* sp. YR343 expressing GFP (YR343-GFP) was cultured in R2A-agar media (pH: 7.2 ± 0.2, Thermo Fisher) at 28°C for 24 hr. For liquid culture studies, single colonies were picked using sterile inoculation loops and mixed in 2 mL R2A broth media (pH: 7.2 ± 0.2, Teknova) supplemented with Kanamycin (150 µg/mL) in sterile test tubes and cultured for 24 hrs (28°C, 215 rpm). To measure growth parameters of YR343-GFP, liquid cultures were diluted to OD_{600} = 0.1 and 100µL was added to a 96 well plate (28°C, 600 rpm) and absorbance (OD_{600}) readings were taken every 10 min. For YR343 monoculture, a lag phase of 2.5 hr and growth rate of 0.673 hr^{-1} were measured, the culture reached stationary phase at 8 hr.

3 16S rRNA community analysis of *Populus trichocarpa* rhizosphere and input samples

The purified gDNAs of the *Populus* rhizosphere enriched sample and the microarray input cultured in R2A media were extracted using E.Z.N.A soil DNA kit (Omega Bio-tek, Norcross, GA), diluted to 20 ng/µl in 100 µL aliquots and sent to Integrated Genomics Facility (Department of Plant Pathology, Kansas State University, Manhattan, KS) for the 16S Illumina sequencing of the hypervariable V3 and V4 region using Nextera XT index Kit v2 (Illumina, Inc., San Diego, CA). 16S rRNA community analysis was performed with Qiime2-2020.8 (Bolyen et al., 2019). The multiplexed raw sequence data with the barcodes were demultiplexed using q2-demux plugin, quality filtered and denoised with q2-dada2 (Callahan et al., 2016) plugin and aligned with mafft (Katoh et al., 2002). The q2-diversity plugin was used to determine alpha-diversity metrics (observed OTUs (DeSantis et al., 2006) and Shannon's diversity index (Shannon, 1948) after rarefaction of the samples to 900 sequences per sample. Taxonomy was assigned to amplicon sequence variants using the silva-138-99-515-806 (Quast et al., 2013) classify-sklearn naïve Bayes taxonomy classifier against the Silva 138 99% OTUs reference sequences (Yilmaz et al., 2014). This analysis found the *Populus* rhizosphere enriched sample and the microarray input cultured in R2A media to contain 120 and 85 observed OTUs and to have Shannon's diversity indices of 4.165 and 3.57, respectively. The q2-taxa plugin was used to explore and visualize the taxonomic composition of the classified sequences by creating taxa bar plots (Bokulich et al., 2018) (**Supplementary Figure 1**). Raw 16S Illumina sequences were uploaded to NCBI sequence read archive (NCBI Accession no. SAMN16795465 and SAMN16795466 for the *P. trichocarpa* rhizosphere-enriched sample and the microarray input respectively).

4 Fabrication of LB-Agar coated PDMS coverslips

To prepare LB-agar coated PDMS coverslips, a modified procedure from Hansen *et al.* was followed (Hansen et al., 2016). 21 g PDMS monomer and 3 g of curing agent were mixed for 3 min, degassed for 30 min, then placed in a 6 in. diameter polystyrene petri dish, degassed again for 30 min and baked at 80°C for 2 hr. PDMS coverslips were ~2000 µm thick. Sterile 25×75mm PDMS coverslips were then cut from the dish, placed in a second polystyrene dish and coated with LB agar by evenly pouring 3 mL of boiling LB agar over the coverslips and the dish. The dish was cooled at 4°C for 30 min to allow the media to solidify over the coverslips and to ensure minimum dehydration. The thickness of the agar layer was ~100 µm. After seeding the microwell substrate with bacteria, microwells were immediately sealed with the coverslips and placed in the humidified, live cell incubator chamber to conduct growth experiments (**Supplementary Figures 4 and 5**).

5 Photodegradable membrane attachment

The procedure for attaching the photodegradable hydrogel membranes to microwell arrays is described in van der Vlies *et al.* (Van Der Vlies et al., 2019) was used here. 25×25 mm clean glass slides (Fisher Scientific) were first functionalized with a non-reactive silane layer by incubating in 20 mL of 0.5% v/v trichloro(1H,1H,2H,2H-perfluorooctyl) silane in toluene for 180 min (Van Der Vlies et al., 2019). Phosphate buffer saline (PBS) with LB solution was then prepared by adding NaH_2PO_4 to LB liquid media to reach 100mM final phosphate concentration and adjusting to pH 8 by adding 5M NaOH (aq.). The membrane precursor solution was then obtained by mixing 12.5 µL of PBS-LB solution with 5.6 µL of photodegradable PEG diacrylate monomer (MW 3400) and 6.9 µL of a four-arm PEG thiol solution monomer (Tibbitt et al., 2013) (MW 10000, NOF America Corporation, DE-100SH). The concentrations of both PEG diacrylate monomer and four-arm PEG thiol solution monomer were 22mM in the precursor solution. 15µL of the liquid pre-cursor solution was then quickly pipetted on top of the perfluoroalkylated glass slides, and the solution was placed over a seeded microwell substrate. Metal spacers were used to provide a constant 38 µm gap between the glass slide and the microwell for the precursor solution. The substrate was then incubated for 25 min at room temperature to allow for membrane formation though monomer crosslinking and gelation (Van Der Vlies et al., 2019). The glass slide was then carefully separated from the membrane-functionalized microwell array and the microwell array was placed on top of a PDMS coverslip and added to the 3D printed scaffold for culture and imaging.

6 Fabrication of 3D printed scaffolds

A custom 3D printed scaffold was designed for imaging microwells with time lapse fluorescence microscopy (TLFM). The 3D printed nylon scaffold was designed using Blender software (Coakley et al., 2014). A seeded and membrane-functionalized microwell was placed in the scaffold, the scaffold was then placed over a standard glass microscope slide. The scaffold holds the sealed microwell substrate approximately 100 µm above the slide surface, as shown in **Supplementary Figure 5**. This allows for bacteria within the wells to receive nutrients and allows the microwell substrate to remain fixed within the focal plane of the 20× objective, eliminating drift in the x,y, and z-directions during the culture period.

7 Membrane degradation and well extraction

Extraction of cell aggregates from microwells followed the protocol recently described in van der Vlies *et al.*(Van Der Vlies et al., 2019). An Olympus BX51 upright microscope equipped with an Infinity 3-1 microscopy camera (Lumenera) and Infinity Analyze software was used to identify the location of target wells according to the on-chip well address. Greyscale images of targeted microwells were taken during extraction with a 20×/0.5NA objective. The Polygon400 photo-patterning instrument (Mightex) containing a 365 nm high-power LED source (50 W) was used to project UV light patterns onto the target well locations. The instrument was attached to the BX51 microscope though an adapter containing dichoic filter cube. A BioLED light source control module equipped with a BioLED analog and digital I/O control module was used to control the light source and a liquid light guide was used to deliver light to the Polygon400. Prior to extraction, the Polygon400 was calibrated using PolyScan2 software and a calibration mirror. For extraction, a cultured microwell array substrate with the attached membrane was first submerged in 1mL R2A broth media to prevent membrane dehydration, then placed under the microscope. PolyScan 2 software was then used to define the irradiation pattern, light intensity and irradiation time. Here, a 165×295µm rectangular working area was defined to accommodate an array of microwells. After a targeted microwell was located, it was exposed a 20 µm diameter circular pattern (20 mW/mm^2, 10 min) to erode the polymer matrix over the well. The opened microwell array was then washed with R2A broth media + 0.05% Tween20 (5×2mL) to extract cells from the opened microwell. The 10 mL wash solution was centrifuged at 2000g for 10 min and the supernatant was carefully removed leaving approximately 2 mL of solution inside the culture tube.

8 96-well plate validation

Separate CFCF from all 5 isolates were mixed together at equal volumes then added to *Pantoea* cell culture in R2A media at a volumetric ratio of 1:1 to reach an OD_{600} value of 0.1. 100µL of each treatment with 6 independent replicates were cultured overnight in 96 well plates to determine the influence of outlier isolates on the OD_{600} of *Pantoea* sp. YR343 growth. Wilcoxon Two-sample tests (Nahm, 2016) were conducted to test whether there is a significant difference between median values of isolate-*Pantoea* combinations and *Pantoea* monoculture.

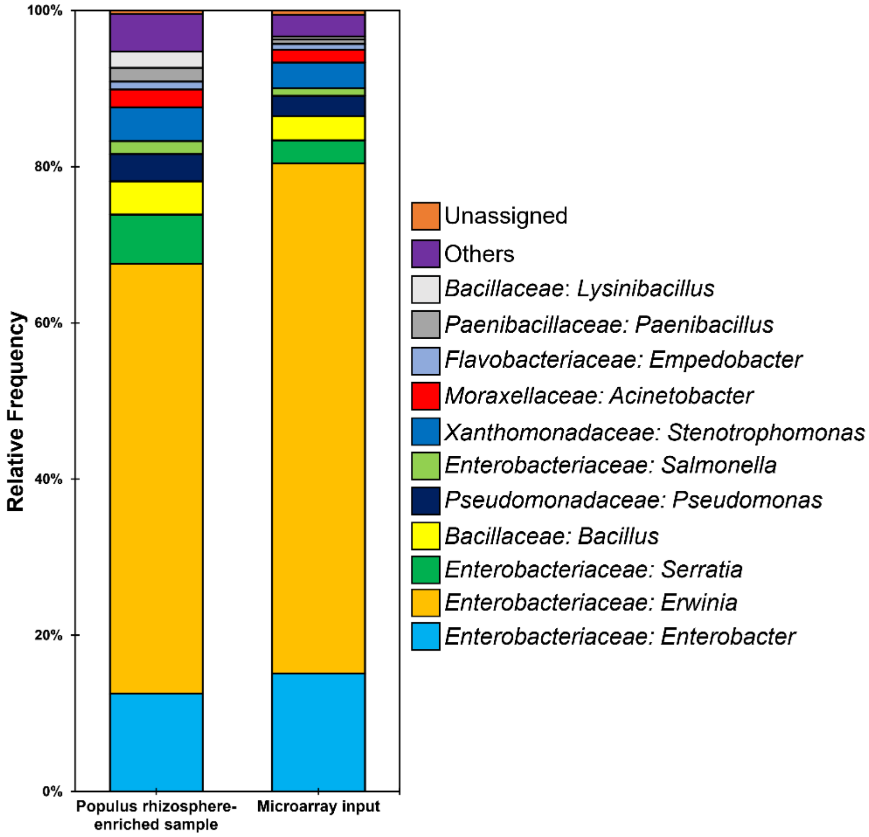

Supplementary Figure 1. Microbial community analysis of 16S rRNA gene sequences of the *P. trichocarpa* rhizosphere-enriched sample (NCBI Accession no. SAMN16795465) and the microarray input (NCBI Accession no. SAMN16795466). The taxa bar chart visualizes the relative abundance of bacterial genera in these samples. Sequences in 99% OTUs were classified and grouped at the genus level. Taxa representing <1% of the total sequences were grouped as Others.

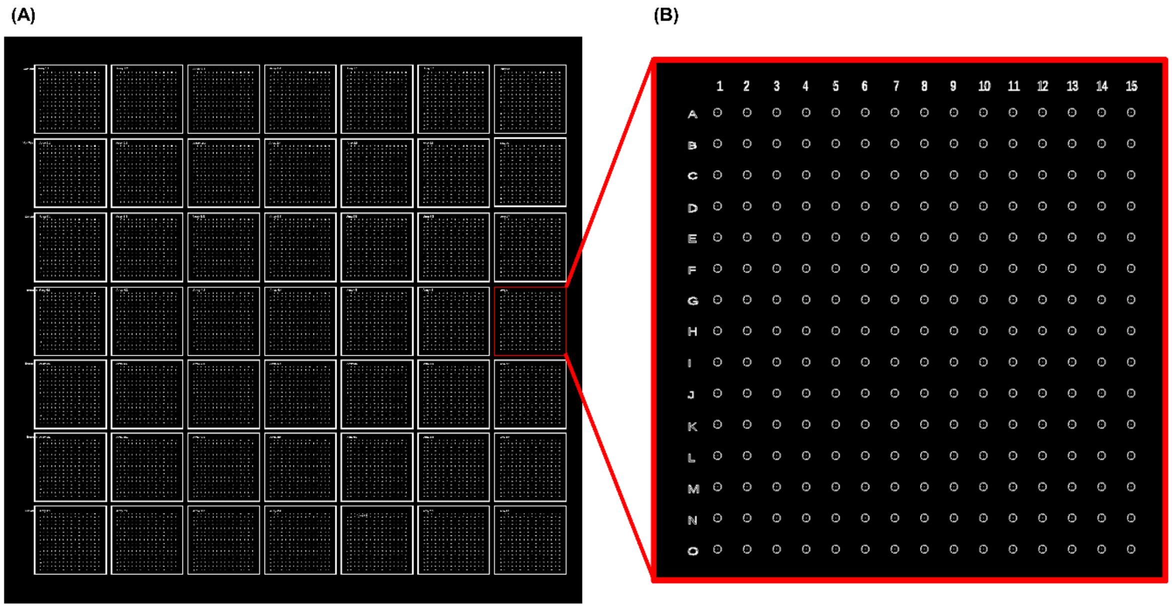

Supplementary Figure 2. MRA layout. (A) The 10µm diameter wells contained 7×7 sub-arrays, each sub-array contained 15×15 arrays of microwells. (B) All wells within the 15×15 sub-array were numbered according to their specific position in the array. Microwells were 10 µm in diameter with a 40 µm pitch.

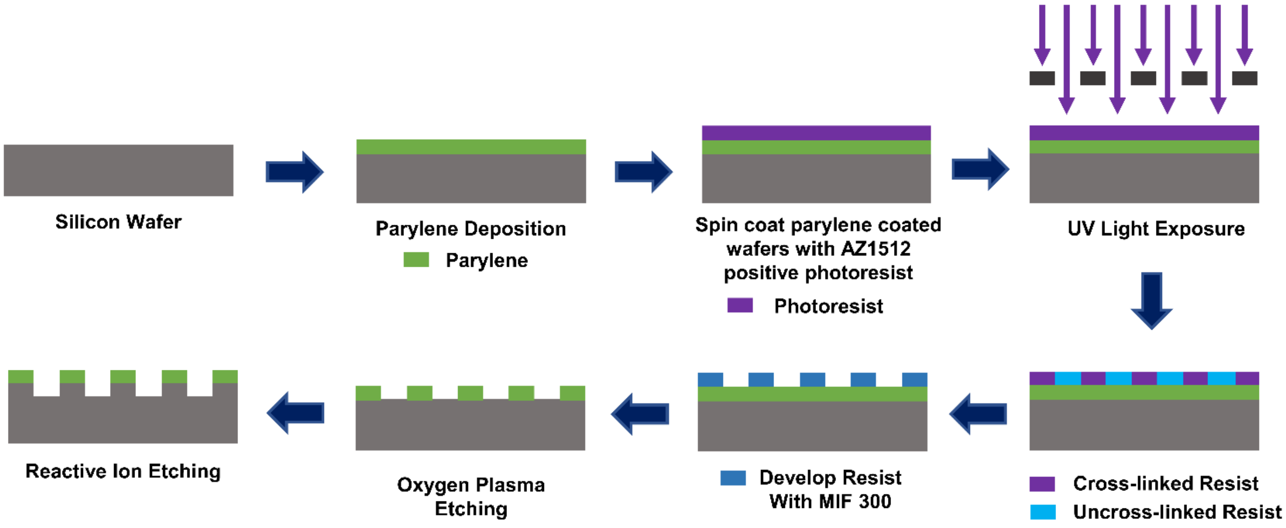

Supplementary Figure 3. Steps for microwell recovery array fabrication. Parylene N was deposited on top of silicon wafers. Then the parylene coated wafers were spin coated with positive photoresist AZ1512 and exposed with UV light though a photomask. The uncrosslinked photoresist was then washed by developing in MIF 300. Then Bosch etching was performed to get MRAs.

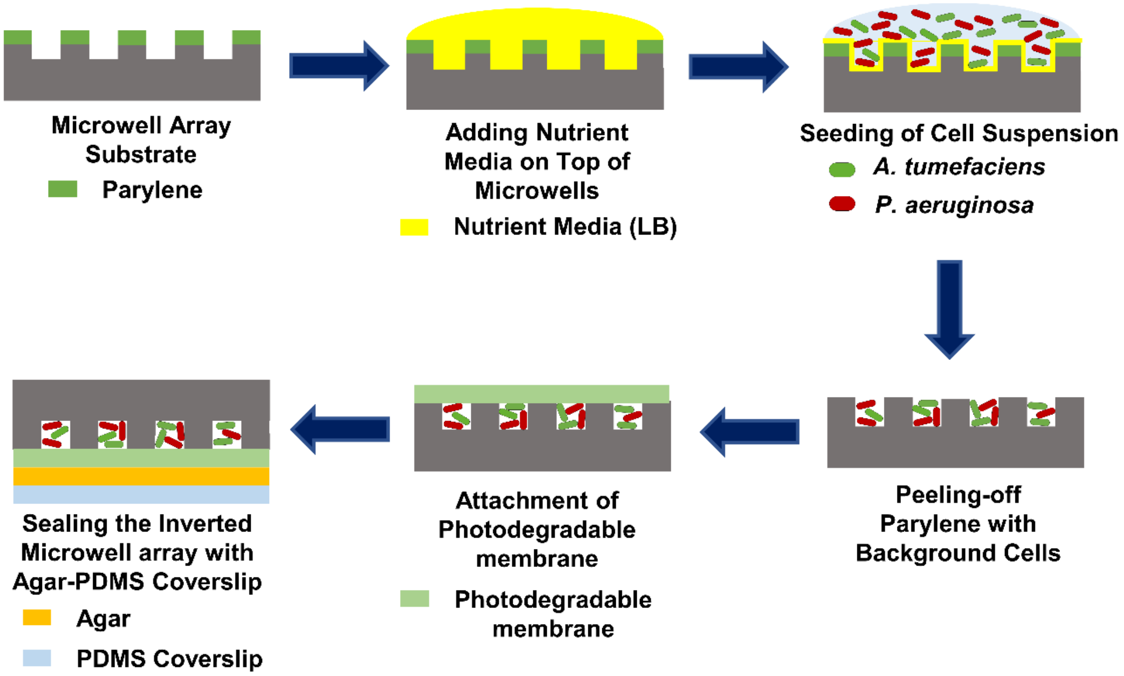

Supplementary Figure 4. Seeding and trapping of bacteria in microwell arrays with the aid of a PDMS coverslip.

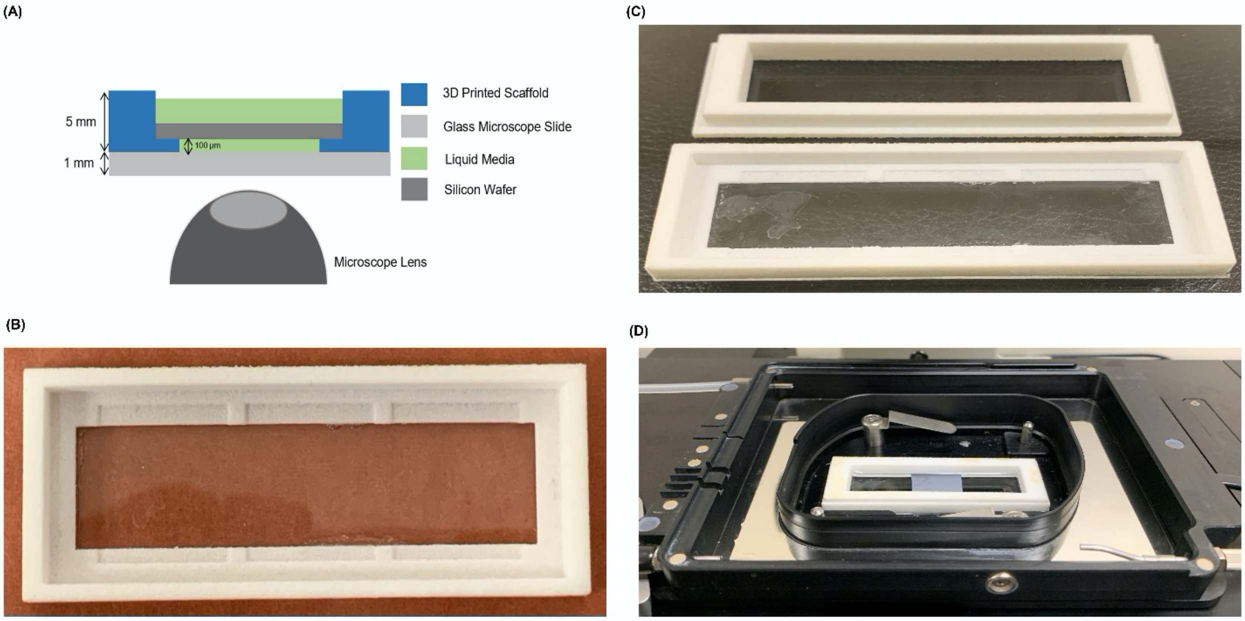

Supplementary Figure 5. (A) Cross section of the 3D printed scaffold on a glass microscope slide. (B) 3D printed Nylon scaffolds. The scaffold had 1.5×1.5cm grooves to hold up to thee microwell arrays. (C) The scaffold was glued to a 75×25mm glass slide then the scaffold lid was attached on top to firmly hold the microwell in place. Liquid nutrient media was then added to fill the space between microwell array and the glass slide, keeping the microwell array fully submerged during culture. (D) The scaffold setup was placed inside a humidified live cell imaging chamber for TLFM.

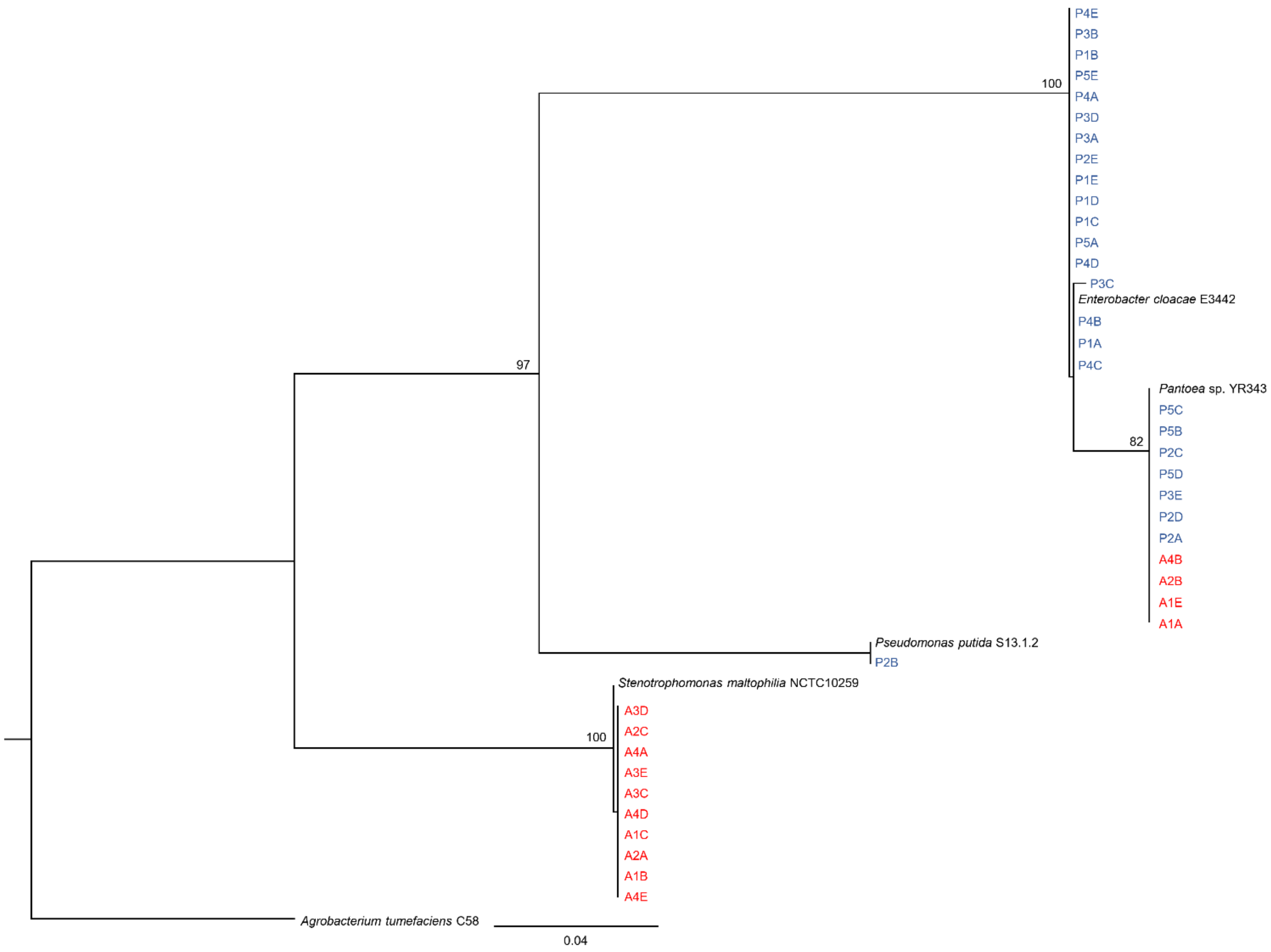

Supplementary Figure 6. Maximum likelihood phylogenetic tree based on partial sequence of the 16S rRNA (1007 bp) from isolates obtained from microwells in which YR343 growth was promoted or antagonized as well as a few reference strains. The tree was constructed using the general time reversible substitution model (Tavaré, 1986) with a gamma distribution (GTR + G) in PhyML 3.3.20190909 (Guindon et al., 2010). We used Smart Model Selection (Lefort et al., 2017) to select this substitution model. Bootstrap values (expressed as a percentage of 1000 replications) higher than 70% are shown at nodes. *A. tumefaciens* C58 was included as an outgroup organism. The partial 16S sequences of the isolates are accessible in GenBank with unique accession numbers (P1E: MW251950, P3C: MW251951, P4B: MW251952, P1D: MW251953, P4A: MW251954, P2E: MW251955, P1B: MW251956, P3A: MW251957, P1C: MW251958, P5E: MW251959, P4C: MW251960, P4D: MW251961, P4E: MW251962, P3B: MW251963, P5A: MW251964, P1A: MW251965, P3D: MW251966, P5C: MW251967, A1A: MW251968, P2D: MW251969, A1E: MW251970, P5B: MW251971, P5D: MW251972, P2A: MW251973, A4B: MW251974, P3E: MW251975, A2B: MW251976, P2C: MW251977, P2B: MW251978, A4E: MW251979, A1B: MW251980, A2C: MW251981, A3D: MW251982, A4A: MW251983, A3E: MW251984, A3C: MW251985, A4D: MW251986, A1C: MW251987, A2A: MW251988).

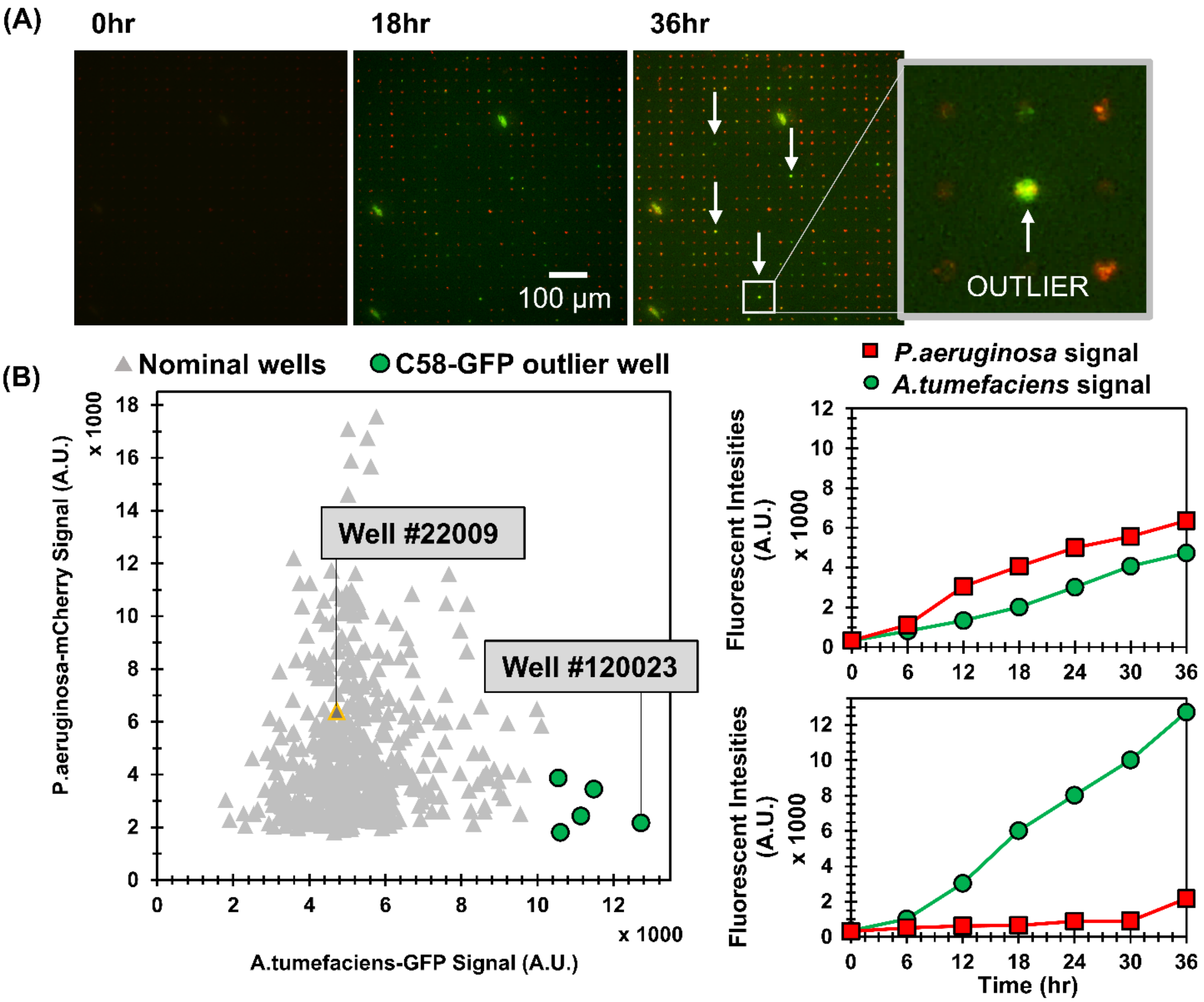

Supplementary Figure 7. C58-GFP - PAO1-mCherry co-culture after seeding at a 1:1 C58:PAO1 cellular ratio. (A) Green-red fluorescence images of co-culture at various time points. (B) Scatter plot of end-point green (C58-GFP) and red (PAO1-mCherry) fluorescent signals (t= 36 hr), C58 outlier wells are identified in green. (C) Individual well growth profile of a nominal well (well #22009 in B) and (D) individual well growth profile of an outlier well (well #120023 in B).

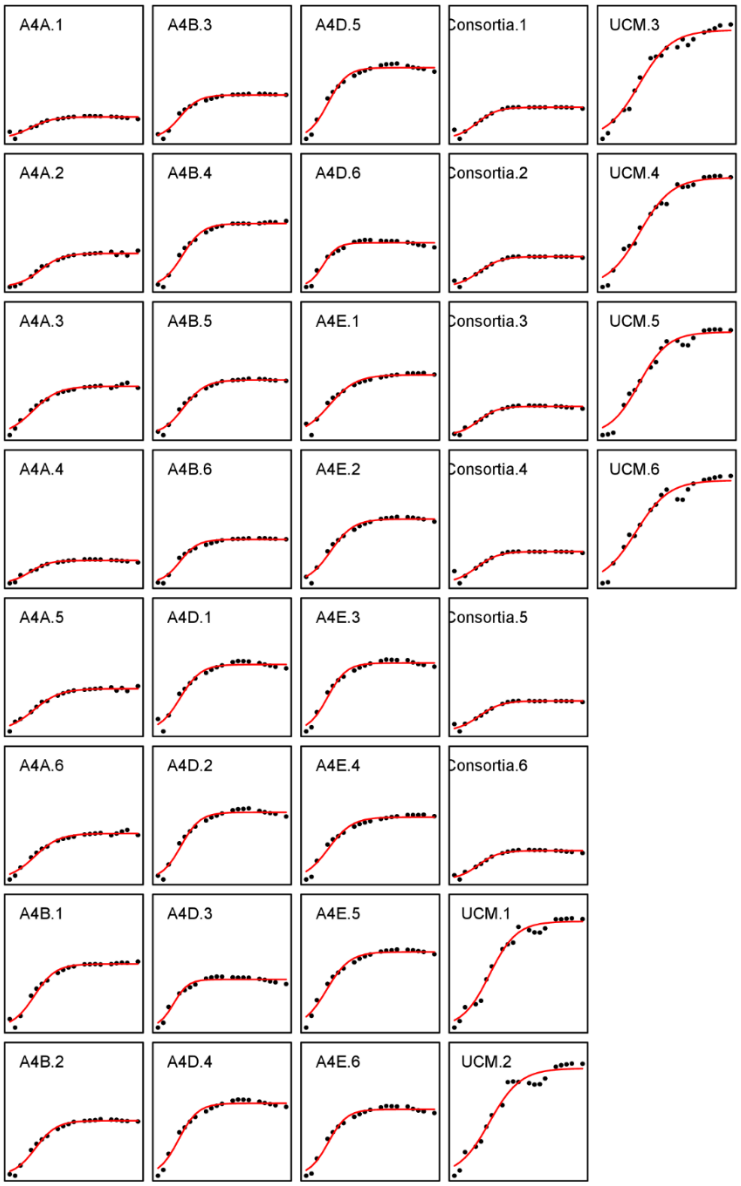

Supplementary Figure 8. Growthcurver output for analysis of growth curves generated from 96 well-plate validation assays using isolates from microwells within which YR343-GFP growth was antagonized. For each condition, YR343-GFP culture was measured for a total of n=6 independent replicates. Carrying capacity, k (OD_{600}) and growth rate, r (h^{-1}) for each isolate, isolate combination and control and were quantified (Sprouffske and Wagner, 2016).

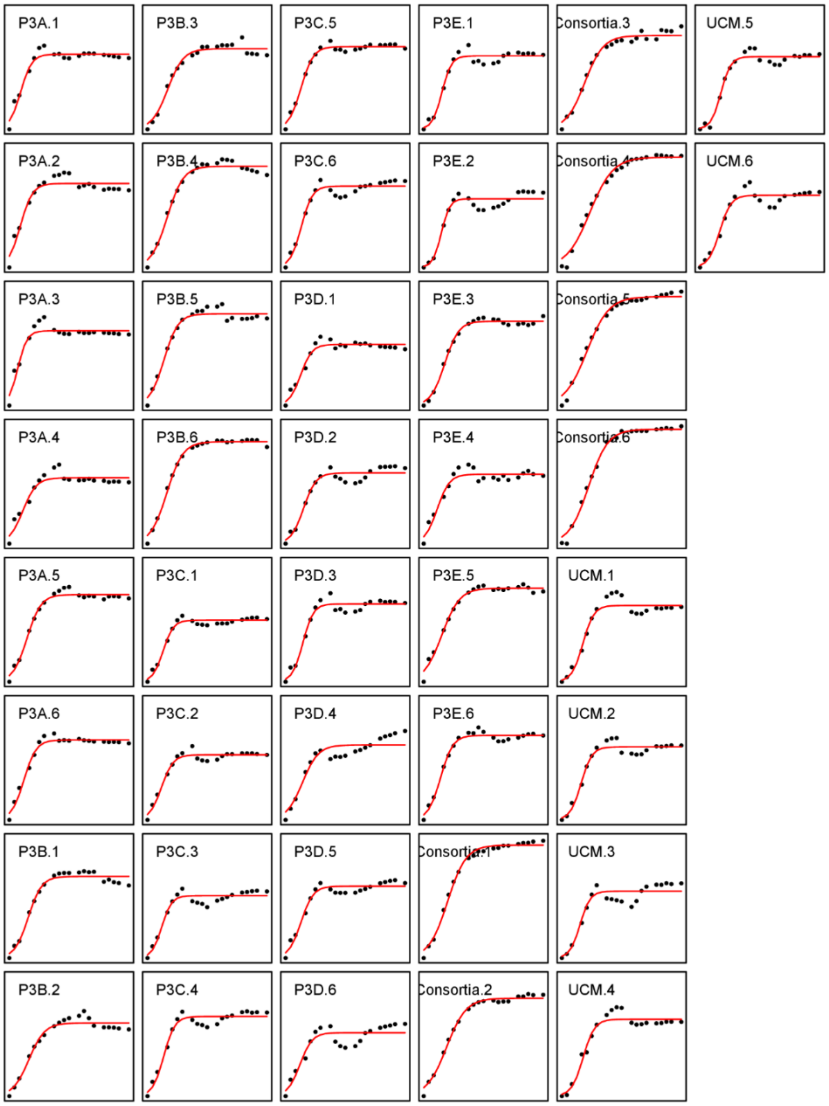

Supplementary Figure 9. Growthcurver output for analysis of growth curves generated from 96 well-plate validation assays using isolates from microwells within which YR343-GFP exhibited promoted population growth. For each condition, YR343-GFP culture was measured for a total of n=6 independent replicates. Carrying capacity, k (OD_{600}) and growth rate, r (h^{-1}) for each isolate, isolate combination and control and were quantified (Sprouffske and Wagner, 2016).

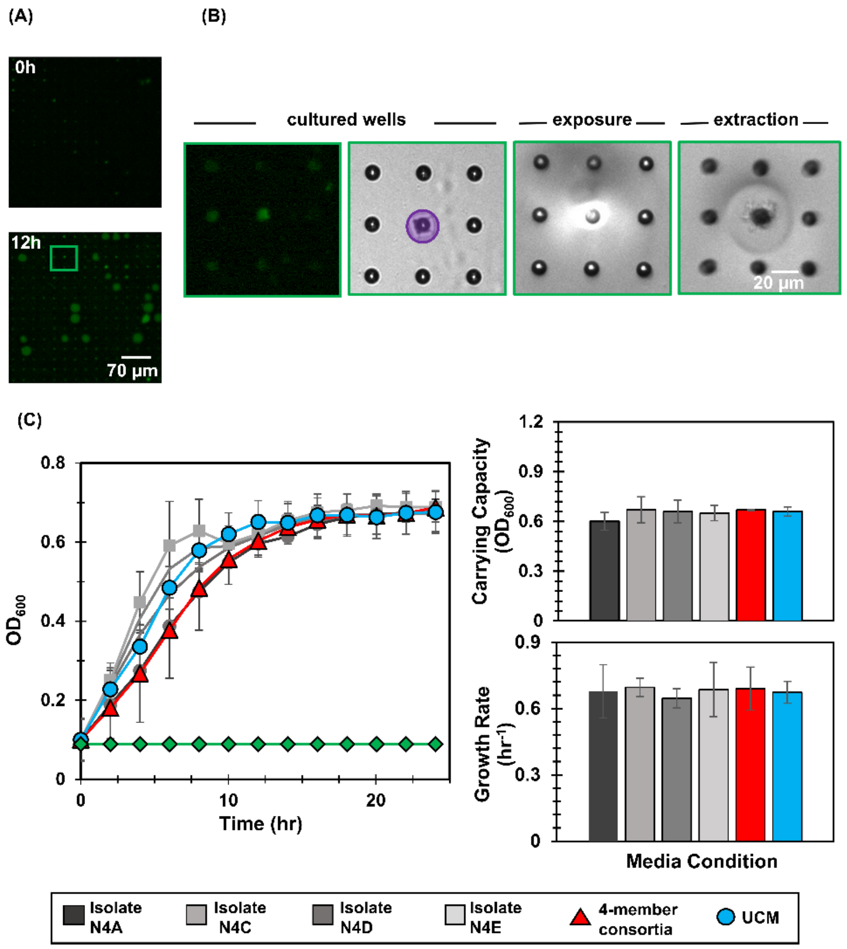

Supplementary Figure 10. Sequential removal of microwells with nominal growth from an array sub-section after co-culture. (A) Microwell array before and after co-culture. This 15×15 microwell array contained wells with no evident increases or decreases in YR343 growth (green). (B) Targeted removal of the microwell community in which YR343 did not see evident increases or decreases in end-point fluorescence (top row, green outline). Purple area denotes UV exposure area used for membrane degradation. (C) Left: YR343 growth curves after inoculation into conditioned media from the nominal isolate, isolate consortia, or unconditioned media (UCM). The control (green line) is conditioned media that was not inoculated with YR343 to verify that there was no growth carry over or contaminating microbes present. Right: Corresponding carrying capacity and growth rates for each growth curve. Statistical differences were identified by comparison of growth metrics between YR343 culture in conditioned media from each isolate or isolate mixture and YR343 growth in UCM (Wilcoxon two-sample test, n=6 independent experiments). None of the isolate mixtures showed significant differences in carrying capacities and growth rates compared to YR343 growth in UCM. These isolates were of Enterobacter genus, identified by 16S amplicon (data not shown).

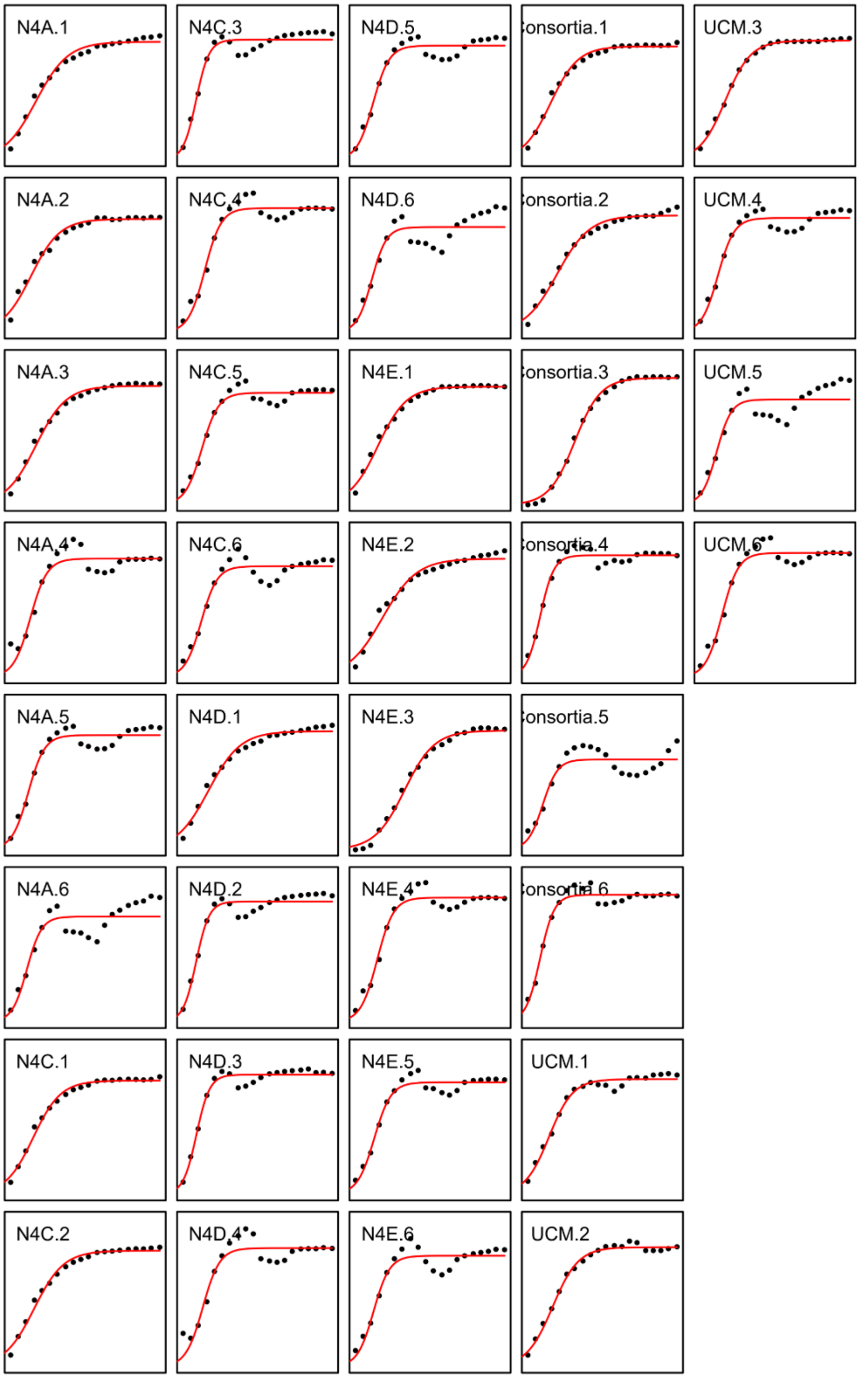

Supplementary Figure 11. Growthcurver output for analysis of growth curves generated from 96 well-plate validation assays using isolates from microwells within which YR343-GFP growth was nominal. For each condition, YR343-GFP culture was measured for a total of n=6 independent replicates. Carrying capacity, k (OD_{600}) and growth rate, r (h^{-1}) for each isolate, isolate combination and control and were quantified (Sprouffske and Wagner, 2016).

Supplementary Table 1. Bacterial strains used in this study.

Strain or Plasmid	Characteristics	Source or reference
**Strains**		
Agrobacterium tumefaciens C58	Wild-type strain	C. Fuqua
A. tumefaciens C58 pSRKKm-sfGFP	Wild-type strain carrying pSRKKm-sfGFP	This study
Pseudomonas aeruginosa PAO1	Wild-type strain	ATCC
P. aeruginosa PAO1 pSRKKm-mCherry	Wild-type strain carrying pSRKKm-mCherry	This study
Pantoea sp. YR343	Wild-type strain with a constitutively expressed chomosomal insertion of EGFP	(Bible et al., 2016)
Escherichia coli S17-1 λpir	λpir, Tra+, cloning strain	(Simon et al., 1983)
E. coli S17-1 λpir pSRKKm-sfGFP	Donor strain carrying pSRKKm-sfGFP	This study
E. coli S17-1 λpir pSRKKm-mCherry	Donor strain carrying pSRKKm-mCherry	This study
P1A	Isolate from microwell P1 within which YR343 exhibited promoted growth	This study
P1B	Isolate from microwell P1	This study
P1C	Isolate from microwell P1	This study
P1D	Isolate from microwell P1	This study
P1E	Isolate from microwell P1	This study

P2A	Isolate from microwell P2 within which YR343 exhibited promoted growth	This study
P2B	Isolate from microwell P2	This study
P2C	Isolate from microwell P2	This study
P2D	Isolate from microwell P2	This study
P2E	Isolate from microwell P2	This study
P3A	Isolate from microwell P3 within which YR343 exhibited promoted growth	This study
P3B	Isolate from microwell P3	This study
P3C	Isolate from microwell P3	This study
P3D	Isolate from microwell P3	This study
P3E	Isolate from microwell P3	This study
P4A	Isolate from microwell P4 within which YR343 exhibited promoted growth	This study
P4B	Isolate from microwell P4	This study
P4C	Isolate from microwell P4	This study
P4D	Isolate from microwell P4	This study
P4E	Isolate from microwell P4	This study
P5A	Isolate from microwell P5 within which YR343 exhibited promoted growth	This study
P5B	Isolate from microwell P5	This study
P5C	Isolate from microwell P5	This study

P5D	Isolate from microwell P5	This study
P5E	Isolate from microwell P5	This study
A1A	Isolate from microwell A1 within which YR343 exhibited antagonized growth	This study
A1B	Isolate from microwell A1	This study
A1C	Isolate from microwell A1	This study
A1D	Isolate from microwell A1	This study
A1E	Isolate from microwell A1	This study
A2A	Isolate from microwell A2 within which YR343 exhibited antagonized growth	This study
A2B	Isolate from microwell A2	This study
A2C	Isolate from microwell A2	This study
A2D	Isolate from microwell A2	This study
A2E	Isolate from microwell A2	This study
A3A	Isolate from microwell A3 within which YR343 exhibited antagonized growth	This study
A3B	Isolate from microwell A3	This study
A3C	Isolate from microwell A3	This study
A3D	Isolate from microwell A3	This study
A3E	Isolate from microwell A3	This study
A4A	Isolate from microwell A4 within which YR343 exhibited antagonized growth	This study

A4B	Isolate from microwell A4	This study
A4C	Isolate from microwell A4	This study
A4D	Isolate from microwell A4	This study
A4E	Isolate from microwell A4	This study
**Plasmids**		
pSRKKm	Broad-host-range P_{lac} expression vector; KmR	(Khan et al., 2008)
pSRKKm-mCherry	IPTG-inducible mCherry expression vector derived from pSRKKm; KmR	(Van Der Vlies et al., 2019)
pSRKKm-sfGFP	IPTG-inducible GFP expression vector derived from pSRKKm; KmR	(Figueroa-Cuilan et al., 2016)

Isolate ID	p-value for Wilcoxon two-sample test	Significance of difference
**A4A**	<0.01	Significant
**A4B**	<0.01	Significant
**A4D**	<0.01	Significant
**A4E**	<0.01	Significant
**4-member consortia**	<0.01	Significant

Supplementary Table 2. Wilcoxon two-sample tests for differences in carrying capacities between YR343-GFP culture in conditioned versus unconditioned media from individual antagonistic isolates or from the 4-membered consortia.

Isolate ID	p-value for Wilcoxon two-sample test	Significance of difference
**A4A**	<0.01	Significant
**A4B**	<0.01	Significant
**A4D**	<0.01	Significant
**A4E**	<0.01	Significant
**4 member consortia**	<0.01	Significant

Supplementary Table 3. Wilcoxon two-sample tests for differences in growth rates between YR343-GFP culture in conditioned versus unconditioned media from individual antagonistic isolates or from the 4-membered consortia.

Isolate ID	p-value for Wilcoxon two-sample test	Significance of difference
**P3A**	0.2876	Not Significant
**P3B**	0.0093	Significant
**P3C**	0.4051	Not Significant
**P3D**	0.0931	Not Significant
**P3E**	0.1149	Not Significant
**5-member consortia**	<0.01	Significant

Supplementary Table 4. Wilcoxon two-sample tests for differences in carrying capacities between YR343-GFP culture in conditioned versus unconditioned media from individual promoting isolates or from the 5-membered consortia.

Isolates ID	p-value for Wilcoxon two-sample test	Significance of difference
**P3A**	0.2876	Not Significant
**P3B**	<0.01	Significant
**P3C**	0.1490	Not Significant
**P3D**	0.0656	Not Significant
**P3E**	<0.01	Significant
**5 member consortia**	<0.01	Significant

Supplementary Table 5. Wilcoxon two-sample tests for differences in growth rates between YR343-GFP culture in conditioned versus unconditioned media from individual promoting isolates or from the 5-membered consortia.

Isolate ID	p-value for Wilcoxon two-sample test	Significance of difference
**N4A**	0.1861	Not Significant
**N4C**	0.4297	Not Significant
**N4D**	0.5249	Not Significant
**N4E**	0.4377	Not Significant
**4-member consortia**	0.3496	Not Significant

Supplementary Table 6. Wilcoxon two-sample tests for differences in carrying capacities between YR343-GFP culture in conditioned versus unconditioned media from individual nominal isolates or from the 4-membered consortia.

Isolate ID	p-value for Wilcoxon two-sample test	Significance of difference
**N4A**	0.2284	Not Significant
**N4C**	0.4870	Not Significant
**N4D**	0.1429	Not Significant
**N4E**	0.1656	Not Significant
**4 member consortia**	0.5314	Not Significant

Supplementary Table 7. Wilcoxon two-sample tests for differences in growth rates between YR343-GFP culture in conditioned versus unconditioned media from individual nominal isolates or from the 4-membered consortia.

9 References

Bible, A. N., Fletcher, S. J., Pelletier, D. A., Schadt, C. W., Jawdy, S. S., Weston, D. J., et al. (2016). A Carotenoid-Deficient Mutant in Pantoea sp. YR343, a Bacteria Isolated from the Rhizosphere of Populus deltoides, Is Defective in Root Colonization. *Frontiers in Microbiology* 7, 491. doi:10.3389/fmicb.2016.00491.

Bolyen, E., Rideout, J. R., Dillon, M. R., Bokulich, N. A., Abnet, C. C., Al-Ghalith, G. A., et al. (2019). Reproducible, interactive, scalable and extensible microbiome data science using QIIME 2. *Nature Biotechnology* 37, 852–857. doi:10.1038/s41587-019-0209-9.

Callahan, B. J., McMurdie, P. J., Rosen, M. J., Han, A. W., Johnson, A. J. A., and Holmes, S. P. (2016). DADA2: High-resolution sample inference from Illumina amplicon data. *Nature Methods* 13, 581–583. doi:10.1038/nmeth.3869.

Coakley, M. F., Hurt, D. E., Weber, N., Mtingwa, M., Fincher, E. C., Alekseyev, V., et al. (2014). The NIH 3D print exchange: A public resource for bioscientific and biomedical 3D prints. *3D Printing and Additive Manufacturing* 1, 137–140. doi:10.1089/3dp.2014.1503.

Figueroa-Cuilan, W., Daniel, J. J., Howell, M., Sulaiman, A., and Brown, P. J. B. (2016). Mini-Tn7 insertion in an artificial attTn7 site enables depletion of the essential master regulator ctrA in the phytopathogen agrobacterium tumefaciens. *Applied and Environmental Microbiology* 82, 5015–5025. doi:10.1128/AEM.01392-16.

Guindon, S., Dufayard, J. F., Lefort, V., Anisimova, M., Hordijk, W., and Gascuel, O. (2010). New algorithms and methods to estimate maximum-likelihood phylogenies: Assessing the performance of PhyML 3.0. *Systematic Biology* 59, 307–321. doi:10.1093/sysbio/syq010.

Hansen, R. H., Timm, A. C., Timm, C. M., Bible, A. N., Morrell-Falvey, J. L., Pelletier, D. A., et al. (2016). Stochastic Assembly of Bacteria in Microwell Arrays Reveals the Importance of Confinement in Community Development. *PLOS ONE* 11, e0155080. doi:10.1371/journal.pone.0155080.

Katoh, K., Misawa, K., Kuma, K. I., and Miyata, T. (2002). MAFFT: A novel method for rapid multiple sequence alignment based on fast Fourier transform. *Nucleic Acids Research* 30, 3059–3066. doi:10.1093/nar/gkf436.

Khan, S. R., Gaines, J., Roop, R. M., and Farrand, S. K. (2008). Broad-host-range expression vectors with tightly regulated promoters and their use to examine the influence of TraR and TraM expression on Ti plasmid quorum sensing. *Applied and Environmental Microbiology* 74, 5053–5062. doi:10.1128/AEM.01098-08.

Lefort, V., Longueville, J. E., and Gascuel, O. (2017). SMS: Smart Model Selection in PhyML. *Molecular biology and evolution* 34, 2422–2424. doi:10.1093/molbev/msx149.

Nahm, F. S. (2016). Nonparametric statistical tests for the continuous data: The basic concept and the practical use. *Korean Journal of Anesthesiology* 69, 8–14. doi:10.4097/kjae.2016.69.1.8.

Price, M. N., Dehal, P. S., and Arkin, A. P. (2010). FastTree 2 - Approximately maximum-likelihood trees for large alignments. *PLoS ONE* 5. doi:10.1371/journal.pone.0009490.

Simon, R., Priefer, U., and Pühler, A. (1983). A Broad Host Range Mobilization System for In Vivo Genetic Engineering: Transposon Mutagenesis in Gram Negative Bacteria. *Bio/Technology* 1, 784–791. doi:10.1038/nbt1183-784.

Sprouffske, K., and Wagner, A. (2016). Growthcurver: An R package for obtaining interpretable metrics from microbial growth curves. *BMC Bioinformatics* 17, 17–20. doi:10.1186/s12859-016-1016-7.

Tavaré, S. (1986). Some probabilistic and statistical problems in the analysis of DNA sequences. *American Mathematical Society: Lectures on Mathematics in the Life Sciences* 17, 57–86. doi:citeulike-article-id:4801403.

Tibbitt, M. W., Kloxin, A. M., Sawicki, L. A., and Anseth, K. S. (2013). Mechanical properties and degradation of chain and step-polymerized photodegradable hydrogels. *Macromolecules* 46, 2785–2792. doi:10.1021/ma302522x.

van der Vlies, A. J., Barua, N., Nieves-Otero, P. A., Platt, T. G., and Hansen, R. R. (2019). On Demand Release and Retrieval of Bacteria from Microwell Arrays Using Photodegradable Hydrogel Membranes. *ACS Applied Bio Materials* 2, 266–276. doi:10.1021/acsabm.8b00592.
